# Copper(II)-Oxyl Formation in a Biomimetic Complex Activated by Hydrogen Peroxide: The Key Role of Trans-Bis(Hydroxo) Species

**DOI:** 10.1021/acs.inorgchem.4c01948

**Published:** 2024-11-25

**Authors:** Ning Cao, Abril C. Castro, David Balcells, Unni Olsbye, Ainara Nova

**Affiliations:** † Department of Chemistry, Centre for Materials and Nanoscience (SMN), University of Oslo, P.O. Box 1033, Blindern, NO-0315 Oslo, Norway; ‡ Hylleraas Centre for Quantum Molecular Sciences, Department of Chemistry, University of Oslo, P.O. Box 1033, Blindern, N-0315 Oslo, Norway

## Abstract

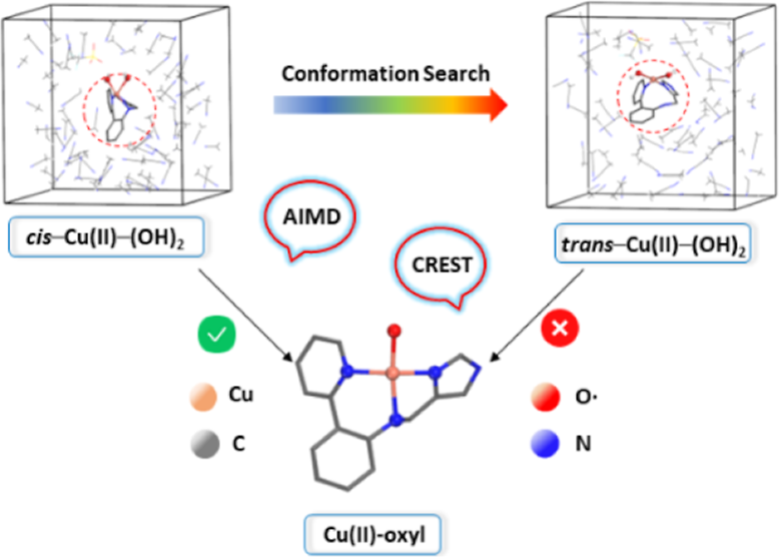

Enzymes in nature, such as the copper-based lytic polysaccharide monooxygenases (LPMOs), have gained significant attention for their exceptional performance in C–H activation reactions. The use of H_2_O_2_ by LPMOs enzymes has also increased the interest in understanding the oxidation mechanism promoted by this oxidant. While some literature proposes Fenton-like chemistry involving the formation of Cu(II)–OH species and the hydroxyl radical, others contend that Cu(I) activation by H_2_O_2_ yields a Cu(II)–oxyl intermediate. In this study, we focused on a bioinspired Cu(I) complex to investigate the reaction mechanism of its oxidation by H_2_O_2_ using density functional theory and ab initio molecular dynamics simulations. The latter approach was found to be critical for finding the key Cu intermediates. Our results show that the highly flexible coordination environment of copper strongly influences the nature of the oxidized Cu(II) species. Furthermore, they suggest the favorable formation of *trans-*Cu(II)–(OH)_2_ moieties in low-coordinated Cu(II) species. This *trans* configuration hinders the formation of Cu(II)–oxyl species, facilitating intramolecular H–abstraction reactions in line with experimentally observed ligand oxidation processes.

## Abstract 2

The formation of Cu-oxyl species using H_2_O_2_ is relevant for understanding the reactivity of Copper-based lytic polysaccharide monooxygenases and designing active catalysts for C–H bond oxidation reactions. This study investigates the formation of Cu-oxyl in a bioinspired *N*,*N*,*N*–Cu(I) complex by density functional theory and ab initio molecular dynamics. Our results highlight the considerable flexibility of the Cu-species and the preferred formation of trans-Cu(II)-(OH)_2_ in low-coordinated sites. This configuration hinders Cu(II)-oxyl formation, favoring ligand oxidation reactions.

## 1 Introduction

**Scheme 1:**
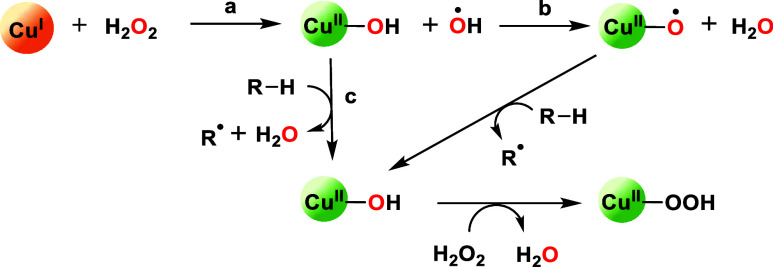
Possible Cu(II) Species Containing O-Bound Ligands Generated by the Oxidation of Cu(I) by H_2_O_2_ (a) Homolytic O–O cleavage to Cu(II)-hydroxo, (b) H-abstraction to Cu(II)–oxyl; and (c) Fenton-like chemistry from •OH radical dissociation.

Lytic polysaccharide monooxygenases (LPMOs) have gained significant attention due to their remarkable ability to catalyze the oxidation of C–H bonds in recalcitrant polysaccharides.^1−5^ Their catalytic prowess stems from a unique feature known as the “histidine brace”, consisting of a mononuclear copper site supported by two imidazole–N ligands from histidine (His) residues and a primary amine.^6−8^ Recent experimental and computational investigations have proposed a peroxygenase cycle in LPMOs, where hydrogen peroxide (H_2_O_2_) reacts with a Cu(I) center, yielding a hydroxyl radical (^•^OH), as shown in Scheme 1a.^3,9−12^ This hydroxyl radical either abstracts a hydrogen atom from Cu(II)–OH, resulting in the formation of Cu(II)–O^•^ (referred to as Cu(II)–oxyl species) and H_2_O (Scheme 1b), or abstracts a hydrogen atom from the substrate (Scheme 1c). In LPMOs, the Cu(II)–oxyl is believed to be a key reactive species for C–H-oxidation based on density functional theory (DFT) calculations.^13−15^ A similar reaction mechanism has also been proposed in biomimetic systems.^16^ In this process, Cu(II)–OH is also present but it is often considered poorly active in C–H activation due to higher H-abstraction energy barriers when compared to the Cu(II)–oxyl. For example, computational studies on methane activation in zeolites reported energy barriers of 26.3 and 11.7 kcal·mol^–1^ for the hydroxyl and oxyl species, respectively.^17,18^ Other Cu(II)-oxidized species, such as Cu(II)–superoxide and Cu(II)–hydroperoxo,^19,20^ can also be generated by the reaction of Cu(I) with H_2_O_2_, but they are usually not active for the oxidation of unactivated C–H bonds. Instead, they are likely to evolve to Cu(II)–oxyl species upon adding protons and electrons from external sources.^21^ In addition, studies done with the [CuO]^+^ ion^22−24^ show that they are active for the C–H oxidation of methane. These studies suggest the importance of Cu(II)–oxyl species in oxidizing C–H bonds, despite direct spectroscopic detection of Cu(II)–oxyl species remains elusive due to their transient nature.

The outstanding C–H activation abilities of LPMOs have inspired researchers to mimic these enzymes using copper complexes and copper metal–organic frameworks.^16,25−30^ For example, Concia et al.^26^ synthesized two Cu(II) complexes with bis(benzimidazole)amine ligands in which Cu–N bond distances in the complexes ranged from ca. 1.97 to 2.03 Å, similar to those observed in the enzymatic systems. The activity of these complexes was tested in the C–H oxidation of *p*-nitrophenyl-β-d-glucopyranoside using H_2_O_2_ as the oxidant, observing a turnover number of 50. Additionally, Ramírez et al. prepared Cu(I) complexes supported by pentadentate nitrogen ligands, proposing that Cu(II)–oxyl species are responsible for the intermolecular C–H activation of pyridylmethyl fragments.^29^ Likewise, Kim et al. also reported a bio-inspired Cu(I) complex in which the N–methyl group of a ligand underwent C–H hydroxylation when H_2_O_2_ was used as the oxidant.^16^

**Scheme 2:**
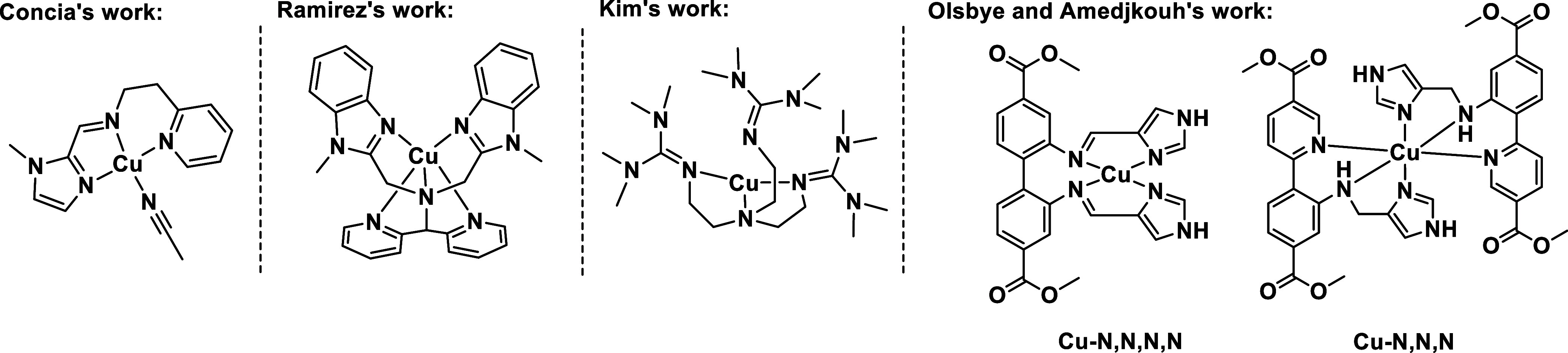
Biomimetic Cu(I) Complexes Showing C–H-Oxidation Reactivity

**Figure 1:**
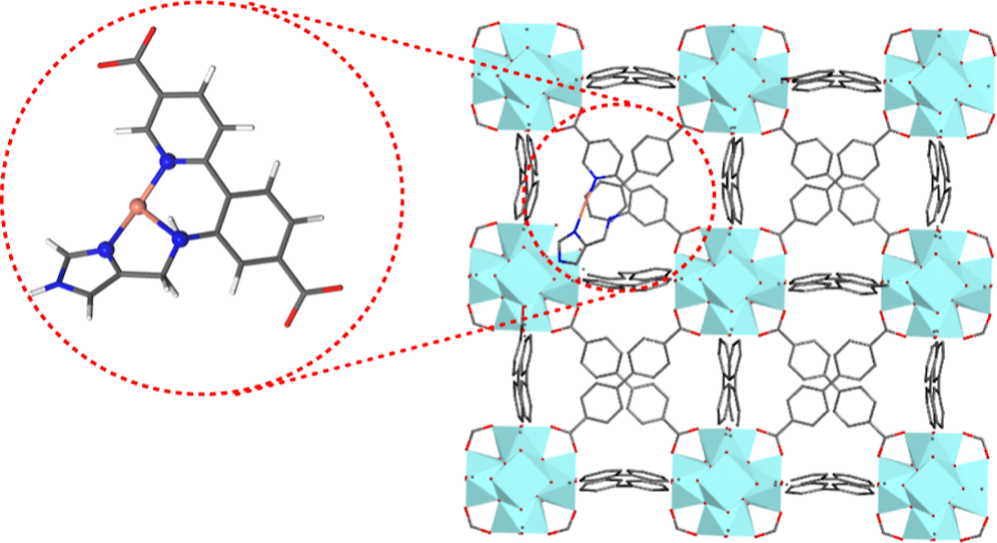
UiO-67 MOF functionalized with the tridentate histidine brace bound to Cu(I) with Zr in turquoise, O in red, C in gray, H in white, N in blue, and Cu in orange.

To mimic the copper active sites within LPMOs, our group recently synthesized a tridentate *N*,*N*,*N*^31^ and a tetradentate *N*,*N*,*N*,*N*^25^ ligand with metal-bound histidine braces (Scheme 2), which were incorporated into the UiO-67 MOF (Figure 1). The characterization of the Cu–N,N,N system in the MOF showed that the metal was ligated by an imidazole-N, a secondary amine, and a pyridine-N. The synthesis of this complex in solution, using esters instead of carboxylate groups, yielded the corresponding CuL_2_ molecular species. The catalytic oxidation of cyclohexane using this Cu–N,N,N complex and H_2_O_2_ in acetonitrile was also evaluated. While 25.6 turnovers were achieved with the complex in solution after 4 h, the MOF showed a smaller turnover number of 0.33 after the same reaction time. These results prompted us to perform a computational study of the underlying reaction mechanism within the MOF to guide further catalyst design efforts.

While several studies have focused on the activation of Cu(I) complexes and LPMOs for C–H oxidation by using DFT methods,^11,16,32,33^ ab initio molecular dynamics (AIMD) simulations have been seldom performed.^34,35^ In this work, AIMD was applied together with static DFT calculations to evaluate the formation of the copper species involved in C–H bond oxidation reactions with the Cu–N,N,N center of the metalated UiO-67 MOF. Our computational results suggest that small structural changes in the flexible coordination sphere of Cu(I)–H_2_O_2_ species trigger major changes in reaction mechanisms, ranging from the direct formation of the Cu(II)–oxyl to its inhibition by a *trans*-Cu(II)–(OH)_2_ intermediate. With these results, we aim to guide experimentalists in devising novel protocols for LPMO-inspired catalysis based on tuning the local environment of the active copper centers.

## 2 Results

**Figure 2:**
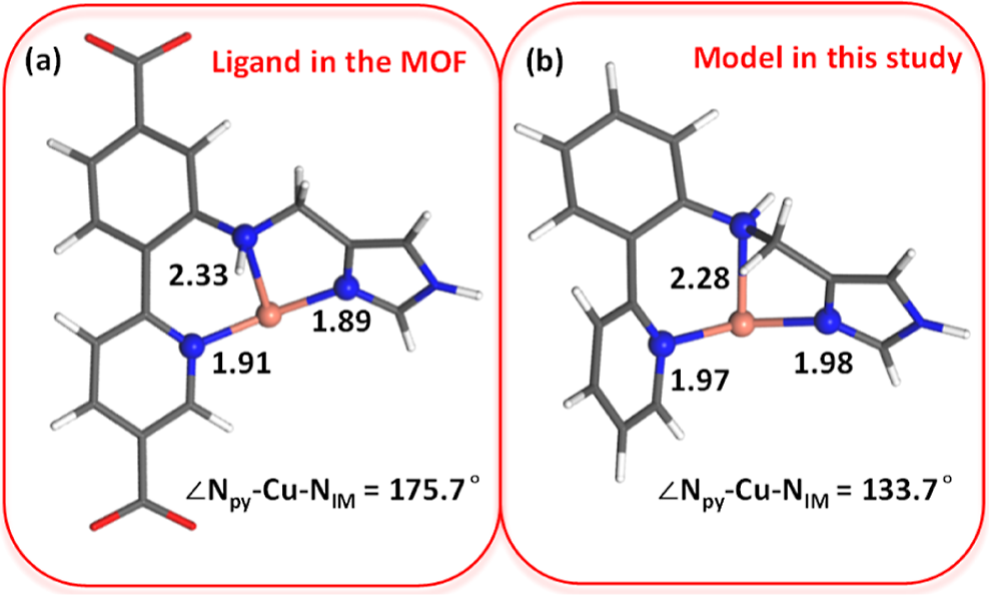
(a) The extracted copper metalated ligand from Cu-UiO-67 MOF as shown in Figure 1. (b) The free cluster model used for calculations in this study. The carboxylate ends were removed, adding protons to balance the charge followed by full optimization. Black labels highlight bond distances (angstrom, Å) in each complex.

To reduce computational cost in AIMD simulations, this study used a simplified model of the Cu complex in the N,N,N–Cu UiO-67 MOF, where the carboxylate groups were replaced with hydrogen atoms and fully optimized. Initially, periodic DFT calculations were carried out to model the Cu–N,N,N UiO-67 MOF structure (see details in the Computational Methods section). The extracted Cu complex from the optimized Cu MOF is depicted in Figure 2a. In this structure, the bond distances of copper with the pyridine-N (Cu–N_py_), the imidazole-N (Cu–N_IM_), and the amine (Cu–N_amine_) ligands were 1.91, 1.89, and 2.33 Å, respectively. The imidazole ring is almost in the same plane as the biphenyl ligand, with a N_py_–Cu–N_IM_ angle of 175.7°. Compared to the Cu-UiO-67 MOF, the free cluster model in Figure 2b is more distorted, as the angle of N_py_–Cu–N_IM_ decreased from 175.7° to 133.7°. Differences in bond distances were also observed, but they were within 0.1 Å. Therefore, we used the small cluster model shown in Figure 2b for the mechanistic study, and the results obtained with the MOF and complex with caboxylates were evaluated afterward (see Section 2.6 and Figure S2).

### 2.1 Static Calculations of the Cu(I) Oxidation by H_2_O_2_

**Figure 3:**
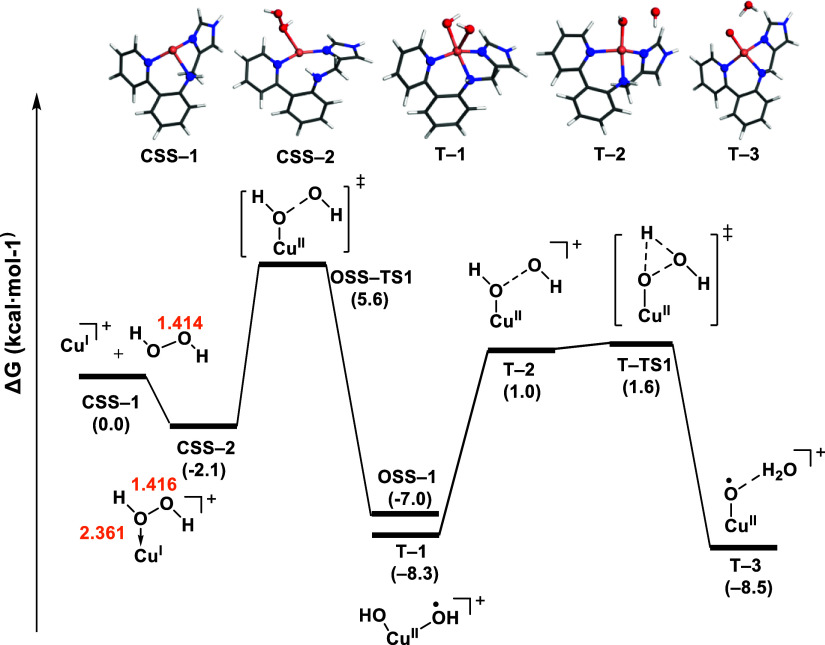
Energy profile for the activation of H_2_O_2_ by the Cu(I) complex in the closed- and open-shell singlet states (**CSS** and **OSS**, respectively), as well as in the triplet state (**T**). The bond distances were labeled in orange.

The activation of H_2_O_2_ by the Cu(I) complex, yielding the Cu(II)–oxyl, was studied using DFT calculations (see Computational Methods section for more details). The reaction starts with the H_2_O_2_ coordination to the closed-shell singlet state, **CSS–1** (Figure 3), as indicated by the orientation of one of the oxygen lone pairs toward Cu. The H_2_O_2_ coordination resulted in the intermediate **CSS–2**, where the O–O bond distance slightly elongates from 1.414 Å (in free H_2_O_2_) to 1.416 Å, and the Cu–O_2_H_2_ bond distance is 2.361 Å. Compared to previous computational studies, this Cu–O bond length is longer than in models of LPMO enzymes with Gln162 and Glu148 residues around Cu (2.10 Å) but shorter than in models where residues have been removed (2.77 Å).^11^ The coordination of H_2_O_2_ to copper in a singlet state configuration has been obtained in a QM/MM study by Wang et al. on LPMO enzymes.^11^ However, other computational studies suggest that the coordination of H_2_O_2_ leads to open-shell species.^12^

The following O–O bond cleavage step involved a low energy barrier of 7.7 kcal·mol^–1^, yielding the dihydroxo intermediate **OSS–1** in the open-shell singlet state, where the hydroxo ligands were bound to the metal center with copper–oxygen bond distances of 1.92 and 2.15 Å, respectively. The triplet state **T–1** (Cu(II)–(OH)_2_) was also considered and it is lower in energy by 1.3 kcal·mol^–1^. The formation of the Cu(II)–oxyl from **T–1** involves the initial dissociation of an OH radical yielding **T–2** (Figure S4), which immediately abstracted one H atom through **T–TS1**, with an energy barrier of 9.9 kcal·mol^–1^, yielding the oxyl and water, in **T–3**. In addition to this pathway, a relaxed scan of the H_2_O_2_ O–O interatomic distance in the unrestricted open-shell singlet and triplet states was also explored, leading to a TS with 4.1 kcal mol^–1^ higher energy than **OSS–TS1**, which was therefore disregarded (Figure S3).

The formation of intermediates similar to **T–1** (Cu(II)–(OH)_2_) and **T–2** (Cu(II)–OH ··· OH) in an enzymatic system has already been reported by Peng et al. using DFT methods.^36^ In particular, they proposed the initial formation of a Cu(II)–OH ··· OH species by homolytic O–O bond cleavage and its subsequent rearrangement to a Cu(II)–(OH)_2_ species with an exergonic energy of −1.5 kcal·mol^–1^. The first formation of **T–1** after the O–O bond cleavage in our system could be due to the lower coordination number (CN = 3) of Cu compared to Peng’s system (CN = 4). This difference together with the larger energy gap between **T–1** and **T–2** made us wonder if other reactivity than OH dissociation could be possible from **T–1**. Therefore, we employed ab initio molecular dynamics (AIMD) simulations to explore the potential energy surface of **T–1**. In particular, we combined a simulated annealing approach^37,38^ with geometry optimizations to identify additional stable conformers of **T–1** (see Computational Methods section).

### 2.2 AIMD of the Cu(II)–(OH)_2_ Intermediate in Acetonitrile

**Figure 4:**
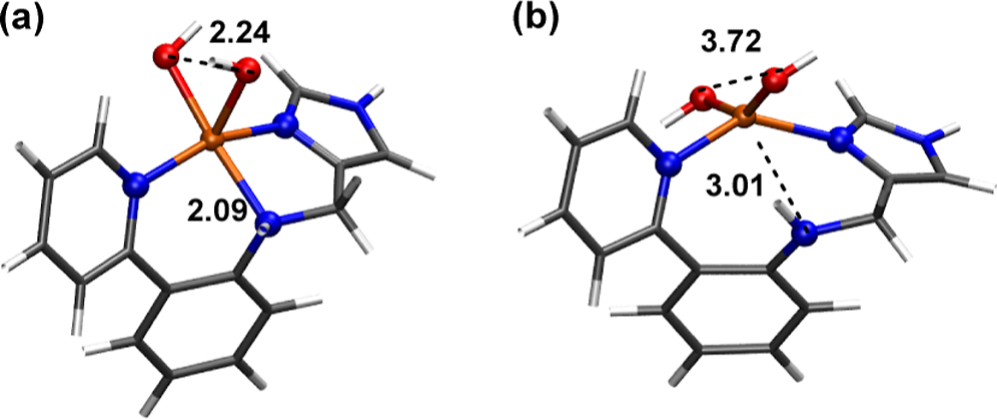
Cu(II)–(OH)_2_ intermediate (**T–1**) in (a) the optimized geometry with Gaussian from the reactivity studies and (b) the abstracted geometry from CP2K after the AIMD simulations.

The AIMD trajectory of intermediate **T–1** (Cu(II)–(OH)_2_) in the *NVT* ensemble with explicit acetonitrile (MeCN) solvation is shown in Figure S5 and Table S1. This trajectory reveals a rapid conversion from the initial geometry to a structure where the amine is no longer coordinated to the copper atom, and the dihydroxo species adopt a *trans* orientation (Figure 4). Hence, the Cu–N_amine_ bond distance increased along the trajectory, extending from 2.09 Å to an average of 3.01 Å. Simultaneously, the O–O interatomic distance underwent a significant elongation, expanding from 2.24 Å to an average of 3.72 Å. The Cu–N_py_ and Cu–N_IM_ bond distances remained relatively stable throughout the trajectory, indicating their robust coordination to the copper center. These values strongly suggest the emergence of a tetradentate copper configuration, implying a significant structural transition. Additionally, we performed a conformational analysis using the Conformer–Rotamer Ensemble Sampling Tool program CREST at the GFN2-xTB semiempirical level^39^ (see Computational Methods for more details). CREST generated a stable conformation that also exhibited an elongation in both the O–O and Cu–N_amine_ interatomic distances (Figure S6), in line with the AIMD results.

The preference for **T–3** over **T–1** from the **T–2** (Cu(II)–OH ··· OH) intermediate (Figure 3) was also investigated using AIMD simulated annealing. We observed the rapid emergence of the Cu(II)–oxyl intermediate within a short time frame (Figure S7), suggesting the preferred formation of the Cu(II)–oxyl species from the Cu(II)–OH ··· OH intermediate, in line to previous studies.^13−15^ This observation suggests that once one of the hydroxo groups decoordinates from copper, the hydrogen atom abstraction occurs immediately, in line with the energy profile shown in Figure 3.

### 2.3 Structure and Reactivity of the *trans*-Cu(II)-(OH)_2_ Complex

**Figure 5:**
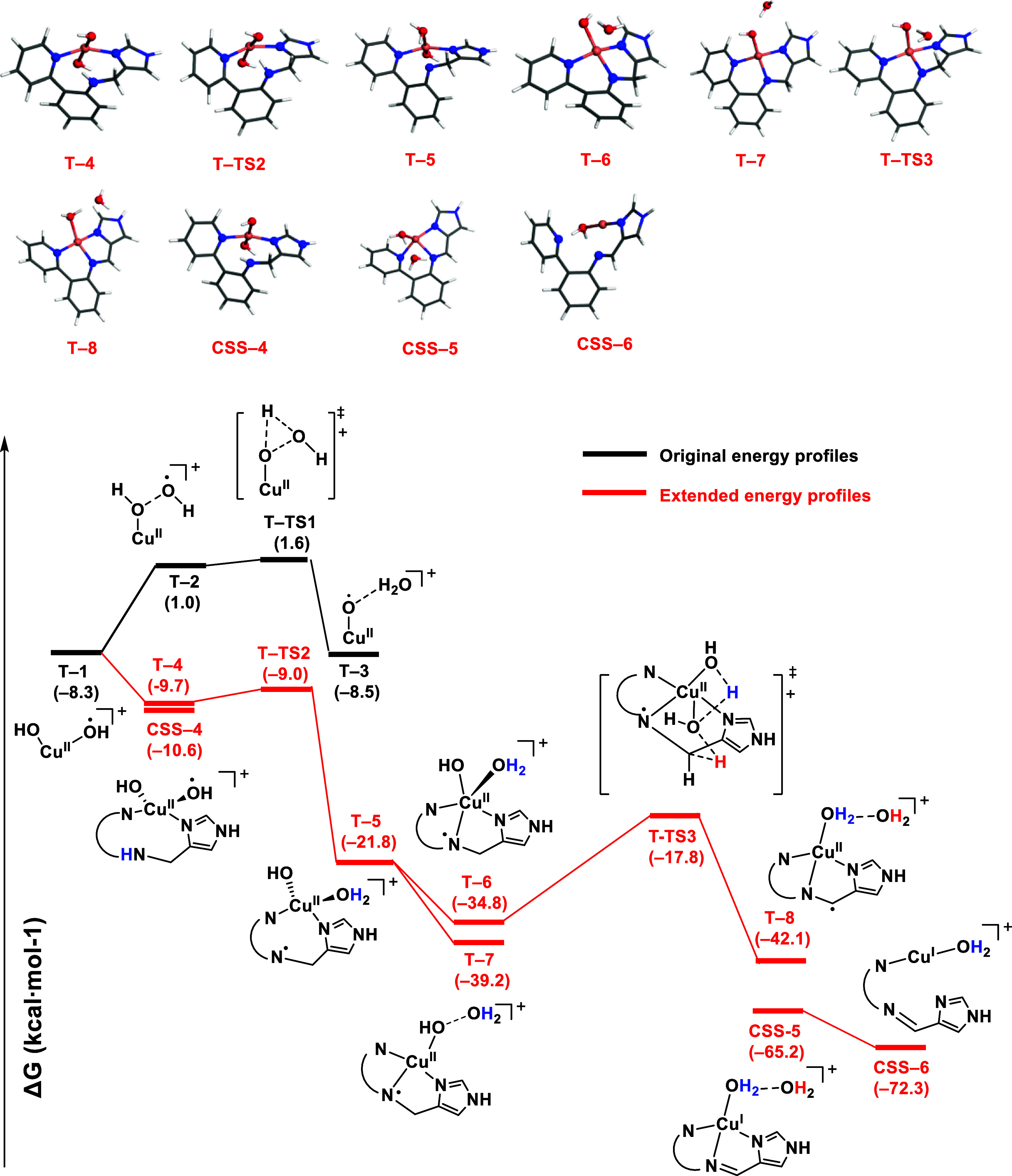
Extended energy profile from the one in Figure 3 (in black) using CCS–1 + H_2_O_2_ as energy reference. In red are structures extracted from AIMD simulations (T–5 and T–6), and a CREST conformational search (T–7) followed by DFT geometry optimization. When the ligand differs from the original, a schematic representation is used.

After finding the novel *trans*-bis-hydroxo intermediate with the AIMD simulations, we used its geometry for further DFT calculations to analyze its structure and reactivity (Figure 5). This species was found to be more stable than the *cis*-bis-hydroxo **T–1** (Cu(II)–(OH)_2_) in both the Cu(III) closed-shell singlet state (**CSS–4**), by 2.3 kcal·mol^–1^, and in the Cu(II) triplet state (**T–4**), by 1.4 kcal·mol^–1^ (comparison between them shown in Figure S8). The time evolution of the spin populations of the Cu atom, O atoms of H_2_O_2_, and N_amine_ atom during the *cis*- to *trans*-Cu(II)-(OH)_2_ isomerization obtained by AIMD simulations was also investigated (see Figure S9). This analysis is consistent with an electron transfer from the N_amine_ atom to the oxygen atoms, contributing to the enhanced stability of the intermediate. In addition, part of the copper electron density is transferred to the hydroxo ligands (see NPA charges in Table S2), making hydroxo ligands more nucleophilic. This facilitates the deprotonation of the amine by a hydroxo ligand in intermediate **T–4** over an energy barrier of only 0.7 kcal·mol^–1^, resulting in the formation of intermediate **T–5**. This transformation was exergonic by −12.1 kcal·mol^–1^, indicating its thermodynamic preference.

Next, we explored the evolution of intermediate **T–5** through AIMD simulations and CREST followed by DFT calculations (Figures S9, S10 and Table S3), which yielded **T–6** and **T–7** intermediates. In **T–6**, the amine coordinates Cu, forming a square-pyramid Cu configuration, which is 13.4 kcal·mol^–1^ below that of **T–5**. Water dissociation in **T–6** is exergonic by 4.0 kcal·mol^–1^, yielding **T–7**, in which the aqua ligand makes an H-bond with the remaining hydroxo ligand. The corresponding closed-shell singlets for these structures were found to be significantly higher in energy (>9 kcal mol^–1^), probably due to the geometry constraints provided by the amido ligand (see Figure S8). Drawing inspiration from the work of Chen et al.,^40^ which describes a similar N radical in an Fe(III) complex, we considered the potential activation of the C–H bond next to the N radical. In the intramolecular hydrogen transfer from **T–6** to **T–8**, which is endergonic by 7.3 kcal·mol^–1^, a hydroxo abstracts a hydrogen atom from the ligand, overcoming an energy barrier of 17.0 kcal·mol^–1^. A water molecule participates in this process, bridging the hydroxo group to the ligand, thereby facilitating the hydrogen transfer. In contrast, the energy barrier of this step is 28.0 kcal·mol^–1^ without the assistance of water (Figure S12). From **T–8**, spin crossover to the closed-shell singlet state is exergonic by −23.1 kcal·mol^–1^ and yields intermediate **CSS–5**. The dehydration of this intermediate is also exergonic, by −7.1 kcal·mol^–1^, and yields intermediate **CCS–6** after decoordination of both the imine and the imidazole ring. Importantly, these results are consistent with experimental observations obtained from NMR studies showing the formation of imine.^26^

### 2.4 Effect of Water Molecules

**Figure 6:**
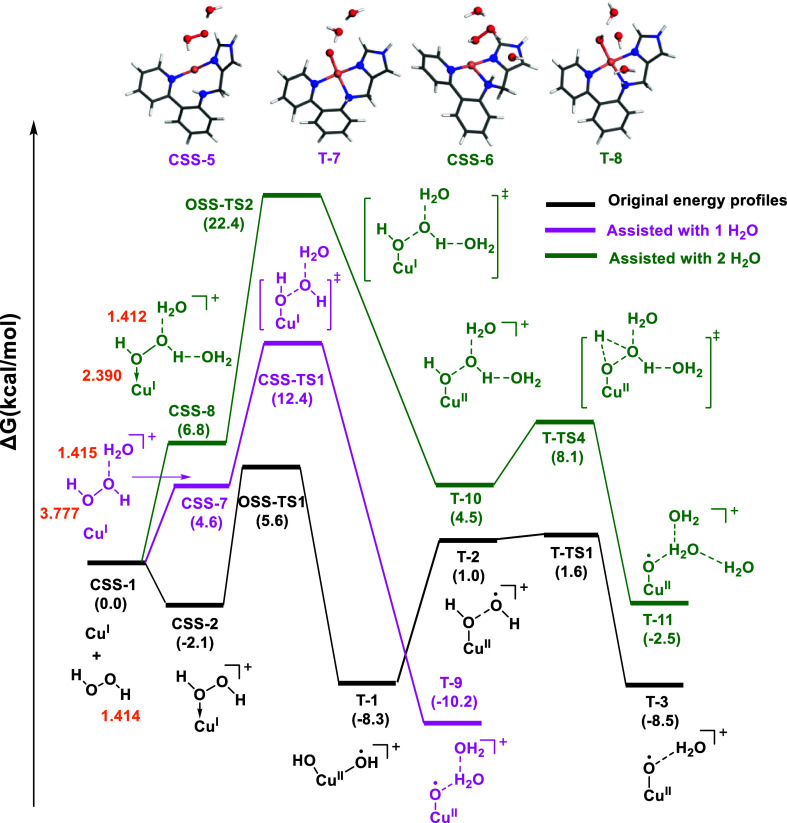
Energy profiles of the Cu(I) complex oxidized by H_2_O_2_ in solvation models including explicit water molecules. The water molecules were manually included in an orientation to facilitate hydrogen bonding with the H_2_O_2_ oxygen and hydrogen. The bond distances were labeled in orange.

To investigate the effect of water on the hydrogen peroxide O–O bond cleavage, we computed the energy profile for the oxidation reaction with one and two explicit water molecules in the calculations (see Figure 6). Despite the solvent used experimentally for the oxidation reaction in the MOF being acetonitrile, commercially available H_2_O_2_ always contains some water to prevent its rapid decomposition into O_2_ and H_2_O. The water molecules were manually included in an orientation to facilitate hydrogen bonding with the H_2_O_2_ oxygen and hydrogen. In **CSS–7**, a water molecule interacts with the oxygen atom in H_2_O_2_ through the hydrogen, while in **CSS–8** and additional water was added to also interact with the hydrogen atom in H_2_O_2_ through the oxygen. The coordination of water to Cu was not considered because of its lower concentration and affinity to Cu compared to acetonitrile (see Figure S13). The introduction of H_2_O molecules did not reduce the energy barrier for O–O cleavage. Instead, it raised the energy barrier from 7.7 to 12.4 and 22.4 kcal·mol^–1^ for one and two additional H_2_O, respectively, compared to the original energy profile (Figure 3). This difference is lower if we consider the unimolecular barriers (7.8 and 15.6 kcal·mol^–1^), which may better represent a high concentration of water. Other ways to correct the large translational entropy contribution resulting from the standard gas-phase approximation can be found in the work of Fang and co-workers and references therein.^41−43^ Furthermore, an intriguing observation emerged when only one H_2_O molecule was introduced: the Cu(II)–oxyl species, **T–9**, can be directly formed after O–O cleavage activation of H_2_O_2_. Additionally, when two H_2_O molecules were present, the intermediate equivalent to **T–1** (Cu(II)–(OH)_2_) did not form. Instead, only one hydroxo species was bound to the copper atom. This result suggests that second-sphere interactions may encumber the formation of Cu(II)–(OH)_2_ bis-hydroxo species promoting the formation of the Cu(II)–oxyl. This will be the most likely scenario in aqueous conditions since these effects seem to be mostly caused by H-bonding.

The influence of two explicit water molecules in intermediate **T–1** (Cu(II)–(OH)_2_) was also evaluated using AIMD simulations. As shown in Figure S14 and Table S4, the O–O distance elongates during the trajectory, ranging from a minimum of 2.52 Å to a maximum of 3.51 Å. This behavior is similar to the system without water. However, the average O–O distance is 3.01 Å when the dihydroxo species interacts with water, which is shorter than in the absence of explicit water (3.72 Å). Additionally, the coordination of the amine group to the copper atom persisted, as evidenced by the average Cu–N_amine_ bond distance of 2.22 Å. Surprisingly, upon reoptimization of the geometry obtained with AIMD, the O–O interatomic distance decreases to 2.77 Å (Figure S15). These results suggest that water stabilizes the hydroxo ligand in an open-shell radical configuration hindering the formation of *trans*-Cu(II)–(OH)_2_. This result was also confirmed by analyzing the time evolution of the spin populations of the Cu atom, O atoms of (OH)_2_, and N_amine_ atom during the AIMD simulations of **T–1**, which showed no significant increase in the spin population of N_amine_ (Figure S16). Further AIMD simulations, including explicit water molecules, were also performed for intermediate **T–10**, resulting in the formation of Cu(II)–oxyl species as observed without water molecules (Figure S17).

### 2.5 Effect of Acetonitrile Coordination

**Figure 7:**
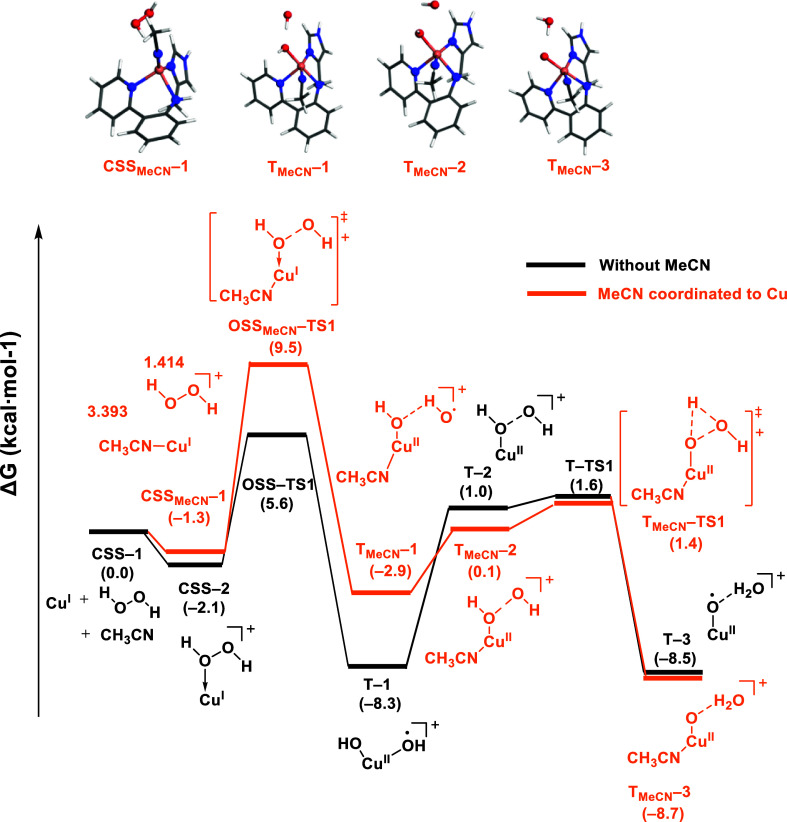
Energy profiles of the MeCN–Cu(I) complex activated by H_2_O_2_. The bond distances were labeled in orange.

We additionally investigated the potential coordination of acetonitrile, a commonly used solvent, as a ligand to the Cu(I) complex (Figure 7). The coordination of acetonitrile to **CSS–1** is exergonic by −1.3 kcal·mol^–1^, yielding the tetracoordinated **CSS**_**MeCN**_**–1** intermediate. Overcoming an energy barrier of 10.8 kcal·mol^–1^, **CSS**_**MeCN**_**–1** transforms into intermediate **T**_**MeCN**_**–1**, where the orientation of one of the hydroxo species differed from **T–2** (Cu(II)–OH ··· OH). In **T**_**MeCN**_**–1**, the hydrogen of one hydroxo pointed toward another hydroxo, forming a distinctive Cu(II)–OH···HO geometry. Subsequently, **T**_**MeCN**_**–1** underwent further rearrangement to form **T**_**MeCN**_**–2** (Cu(II)–OH ··· OH), albeit at an endergonic cost of 3.0 kcal·mol^–1^. The formation of Cu(II)–oxyl was achieved through a transition state, **T**_**MeCN**_**–TS1**, overcoming an energy barrier of 1.3 kcal·mol^–1^. The Cu(II)–OH···HO arrangement bears a resemblance to an iron(IV)–oxo system suggested by Singh et al.,^44^ who proposed an hydrogen atom abstraction as the primary mechanism for oxyl formation.

**Figure 8:**
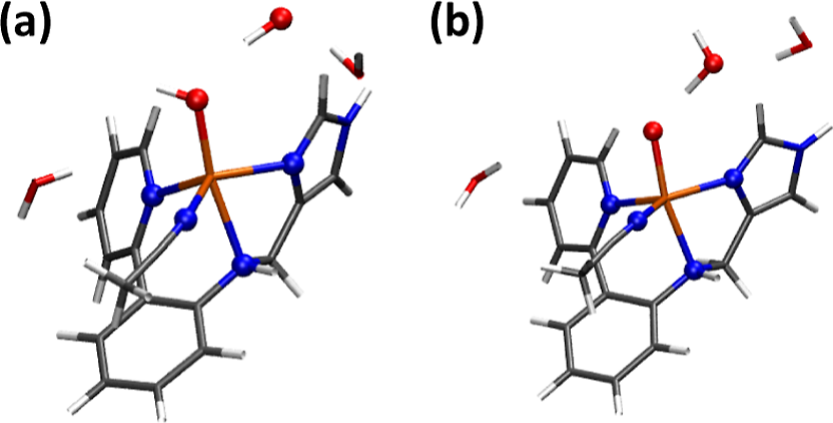
(a) Initial **T**_**MeCN**_**–1** optimized geometry with two water molecules; (b) final **T**_**MeCN**_**–1** geometry after AIMD simulations.

To investigate the ability of **T**_**MeCN**_**–1** intermediate (Cu(II)–OH ··· HO) to form Cu(II)–oxyl or rather yielding OH diffusion into the solution, we used AIMD simulations in explicit acetonitrile and included two water molecules surrounding the hydroxo ligands. As in the case of Cu(II)–OH ··· OH, the formation of Cu(II)–oxyl was observed in a very short time frame (Figure 8b). In an attempt to observe the formation of Cu(II)–oxyl species over an extended duration, we omitted the simulated annealing protocol. In this case, in contrast to with Cu(II)–OH···OH, the Cu(II)–OH···HO geometry remained unchanged after the full NVT trajectory of 20 ps (Figure S18 and Table S5).

**Figure 9:**
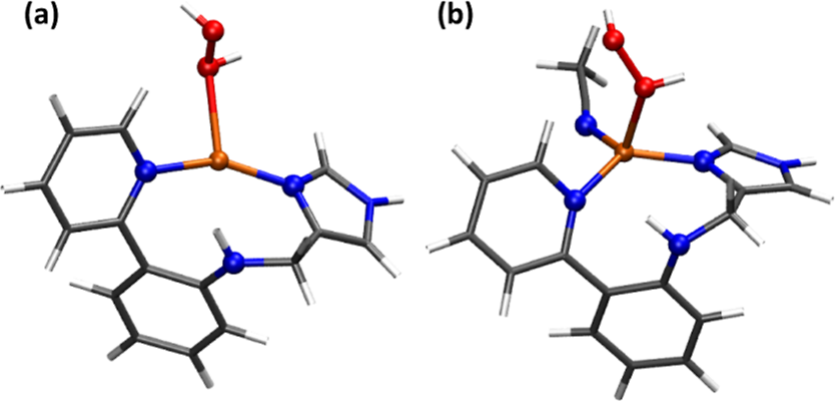
(a) The initial **CSS–2** optimized geometry in Gaussian. (b) The abstracted final geometry located along the NVT trajectory from CP2K.

The energy profile in Figure 7 showed the concerted coordination of H_2_O_2_ and cleavage of the O–O bond, yielding **T**_**MeCN**_**–1** and suggesting the unfavorable coordination of H_2_O_2_ when acetonitrile is bound to copper in **CSS–2**. To corroborate this result, we performed AIMD simulations from **CSS–2** in explicit MeCN solvent (see Figures 9, S19, and Table S6). Throughout these simulations, the Cu–N_py_, Cu–N_IM_, and Cu–N_amine_ bond distances exhibited minimal changes, while one MeCN molecule coordinates to copper. Besides, the Cu–O_2_H_2_ bond distance was elongated by ∼0.2 Å. After observing the coordination of MeCN, the system remained nearly unchanged during the trajectory, which was extended for 20 ps in the *NVT* ensemble. Hence, this result is consistent with a tetra-coordinated copper configuration.

**Figure 10:**
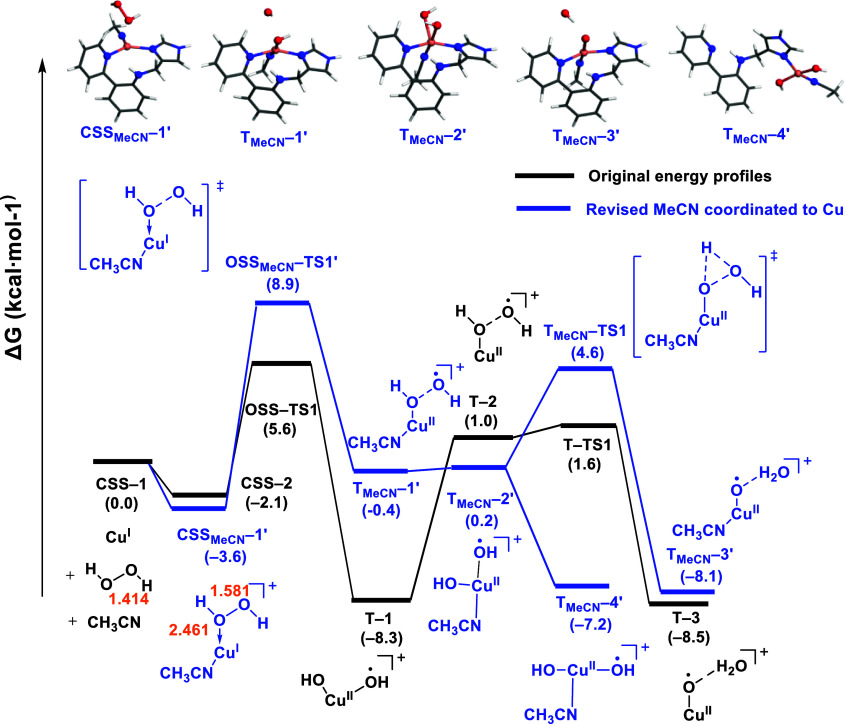
Extended energy profiles in acetonitrile derived from the intermediate discovered in the AIMD simulations. The bond distances were labeled in orange. In blue are structures extracted from AIMD simulations (**CSS**_**MeCN**_**–1′**), and a CREST conformational search (**CSS**_**MeCN**_**–4′**) followed by DFT geometry optimization.

The subsequent reoptimization of the new intermediate obtained through AIMD resulted in the formation of intermediate **CSS**_**MeCN**_**–1′**, which is lower in energy than **CSS–2** by −1.5 kcal·mol^–1^ (Figure 10). Notably, although the distance between H_2_O_2_ and copper is 2.461 Å, the O–O bond distance of H_2_O_2_ is elongated from 1.414 to 1.581 Å, suggesting the activation of H_2_O_2_ by the Cu(I) complex. Proceeding from this intermediate, the O–O bond cleavage of H_2_O_2_ yields the product **T**_**MeCN**_**–1′**, featuring the Cu(II)–OH ··· OH moiety. This intermediate, instead of froming a Cu(II)–oxyl, it transitioned to an intermediate **T**_**MeCN**_**–2′** in which the hydroxo moved back to the Cu site. This step is followed by a hydrogen atom transfer between dihydroxo groups, yielding **T**_**MeCN**_**–3′**. This transition involved overcoming an energy barrier of 4.4 kcal·mol^–1^. Given the previous sections highlighting the preference of the Cu(II)–(OH)_2_ moiety to adopt the *trans* configuration, we conducted a CREST conformational search in **T**_**MeCN**_**–2′**. This was followed by a DFT optimization, resulting in the discovery of a new intermediate: **T**_**MeCN**_**–4′**. In this species, the two hydroxo ligands and the N_IM_ and N_MeCN_ were *trans* to each other, while N_amine_ and N_py_ had decoordinated from copper. As copper was bound to the ligand only through the Cu–N_IM_ bond, this species would not be stable within the MOFs, which is in line with the copper leaching observed experimentally during the reaction.

### 2.6 Comparison between the Cu Cluster Model and the Periodic Cu UiO-67 MOF

**Figure 11:**
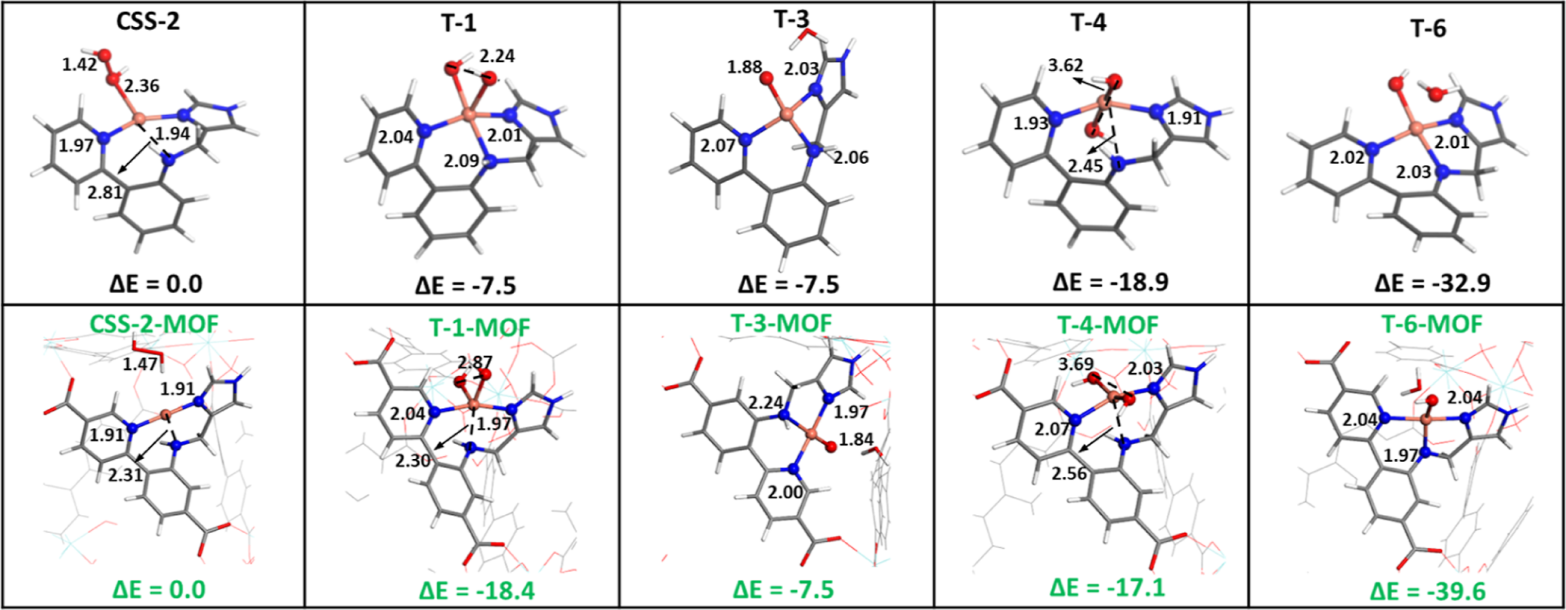
Optimized intermediates of the tridentate Cu complex isolated (top) and within UiO-67 MOF (bottom). The potential energies are shown below each geometry, in kcal mol^–1^. Bond distances are in Å.

To evaluate if our findings using the molecular Cu system can be extrapolated to the Cu UiO-67 MOF, the key intermediates identified with the cluster model were optimized using a periodic model in CP2K. The geometries and potential energies obtained with both systems are shown in Figure 11. Compared to intermediate **CSS–2**, where H_2_O_2_ is coordinated to Cu(I), H_2_O_2_ is far from the copper site in the MOF, with the same O–O bond distance (1.47 Å) observed in free H_2_O_2_. This different behavior can be attributed to the influence of carboxylate groups in the MOF, which increases the energy for H_2_O_2_ coordination to Cu (see Figure S2) and the stabilization of H_2_O_2_ in the pore by weak interactions. Nevertheless, the formation of the *cis*-Cu(II)–(OH)_2_ is exothermic with an energy of −18.4 kcal mol^–1^ (−7.5 for the oxyl). Interestingly, the energies of the *cis*- and *trans*-Cu(II)-(OH)_2_ isomers are very similar in the MOF, with a difference close to 1 kcal mol^–1^. This result suggests that both isomers might be in equilibrium. Nonetheless, the amine hydrogen abstraction by the OH radical remains highly favorable in both the complex and the MOF, and it is preferred over the formation of the Cu-oxyl.

**Figure 12:**
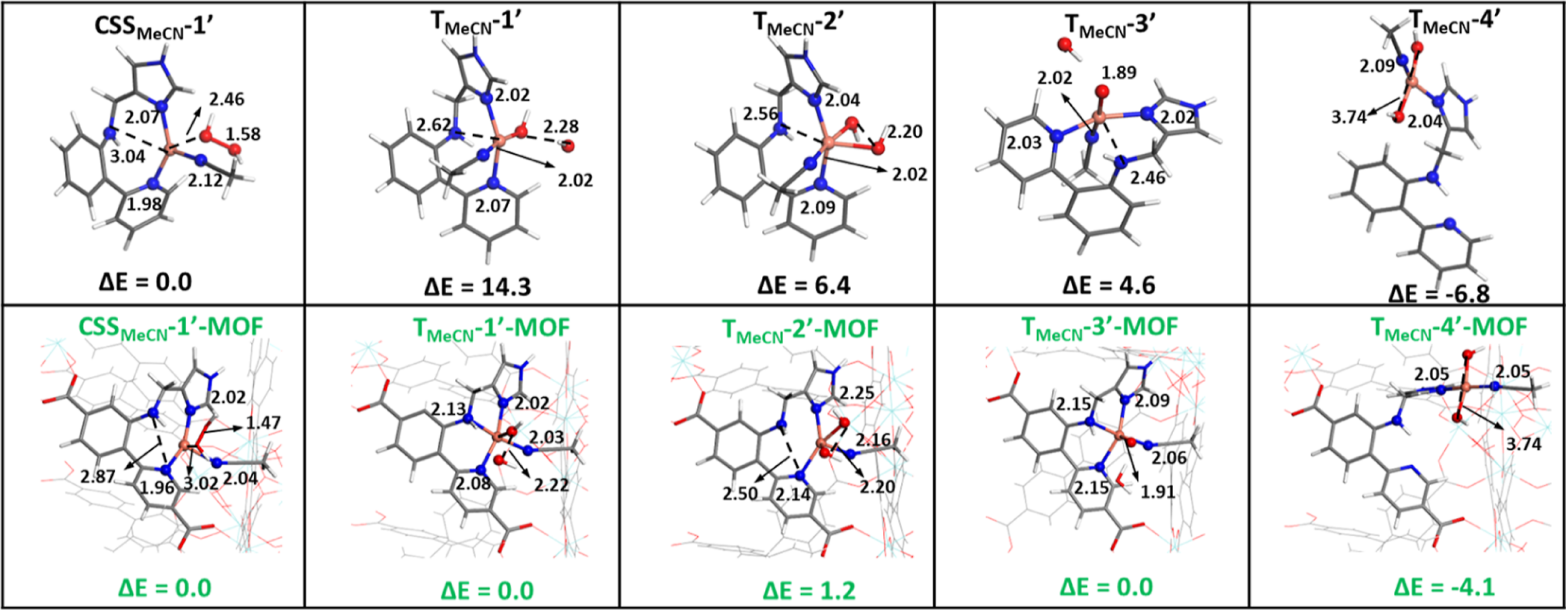
Optimized intermediates of the tetradentate Cu complex and the Cu UiO-67 MOF coordinated with acetonitrile. The potential energies are shown at the bottom right with the unit of kcal mol^–1^. The bond distances are labeled in black with the unit of Å.

We also investigated the acetonitrile-ligated Cu species in the MOF, as depicted in Figure 12. Similar to the system without acetonitrile, the H_2_O_2_ coordination to Cu is weaker in **CSS**_**MeCN**_**-1′-MOF** compared to the isolated Cu complex (**CSS**_**MeCN**_**–1′)**, with a Cu–O distance of 3.02 Å and no change in the O–O bond compared to the free H_2_O_2_ molecule. In addition, the O–O bond cleavage is more favorable in the MOF (Δ*E* = 0 kcal mol^–1^) compared to the molecular system (Δ*E* = +14.3 kcal mol^–1^), yielding intermediate **T**_**MeCN**_**1′-MOF**, which has a similar energy to the *cis-*Cu(OH)_2_ isomer (**T**_**MeCN**_**2′-MOF**). These intermediates can evolve to either the Cu-oxyl intermediate (**T**_**MeCN**_**3′-MOF**) or the *trans*-Cu(OH)_2_ isomer (**T**_**MeCN**_**4′-MOF**). Among these two reactions, the latter is thermodynamically preferred by 4.1 kcal mol^–1^. Overall, these results suggest that the *trans*-Cu(OH)_2_ can also be formed within the Cu UiO-67 MOF and play a critical role in the oxidation of C–H bonds.

## 3 Discussion

**Scheme 3:**
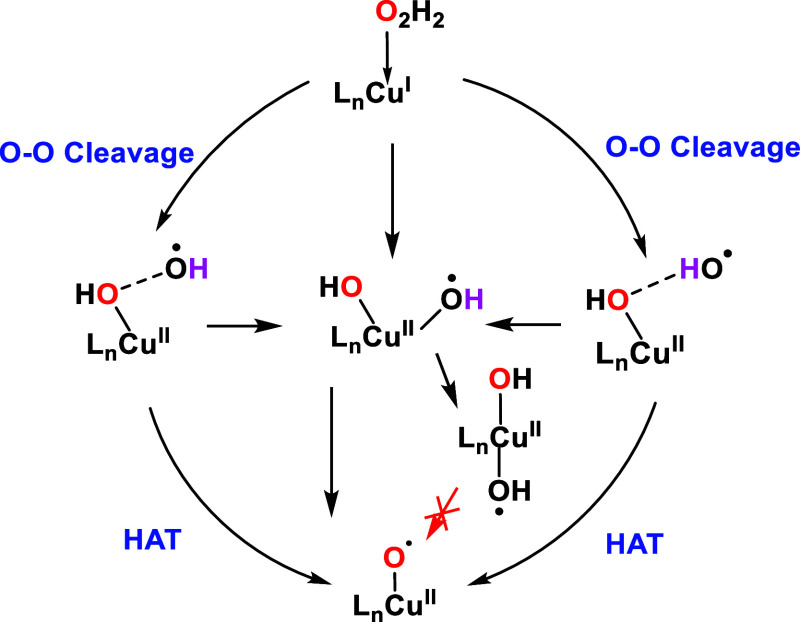
Different Reaction Pathways of the Copper Site Activated by H_2_O_2_ to form Different Cu(II) Oxygen Species

Based on the findings from both static and dynamic calculations, we summarized the different reaction pathways in Scheme 3. The O–O bond cleavage on the copper site can generate a range of Cu(II) oxygen species. Three primary species were observed in our DFT calculations: Cu(II)–(OH)_2_, Cu(II)–OH···HO, and Cu(II)–OH···OH, the latter only differing in the orientation of the OH groups. In our investigation, the formation of Cu(II)–oxyl was favorable when Cu(II)–OH···OH and Cu(II)–OH···HO species were formed, as it requires overcoming a barrierless process. By contrast, Cu(II)–(OH)_2_ has a preference for isomerizing to a *trans* configuration and was unlikely to undergo hydrogen atom transfer to form a Cu(II)–oxyl. These three Cu(II) oxygen species are consistent with previous research by Peng et al.,^36^ who reported them in the H_2_O_2_ activation mechanism at the Cu_C_ site of pMMOs. In contrast to our results, they proposed that even when Cu(II)–(OH)_2_ species are present, Cu(II)–oxyl can still form through HAT between the dihydroxo species. To understand this difference, we conducted a conformational search of Cu(II)–(OH)_2_, aiming to determine its propensity for undergoing *trans* isomerization when copper coordinates to other different ligands.

**Figure 13:**
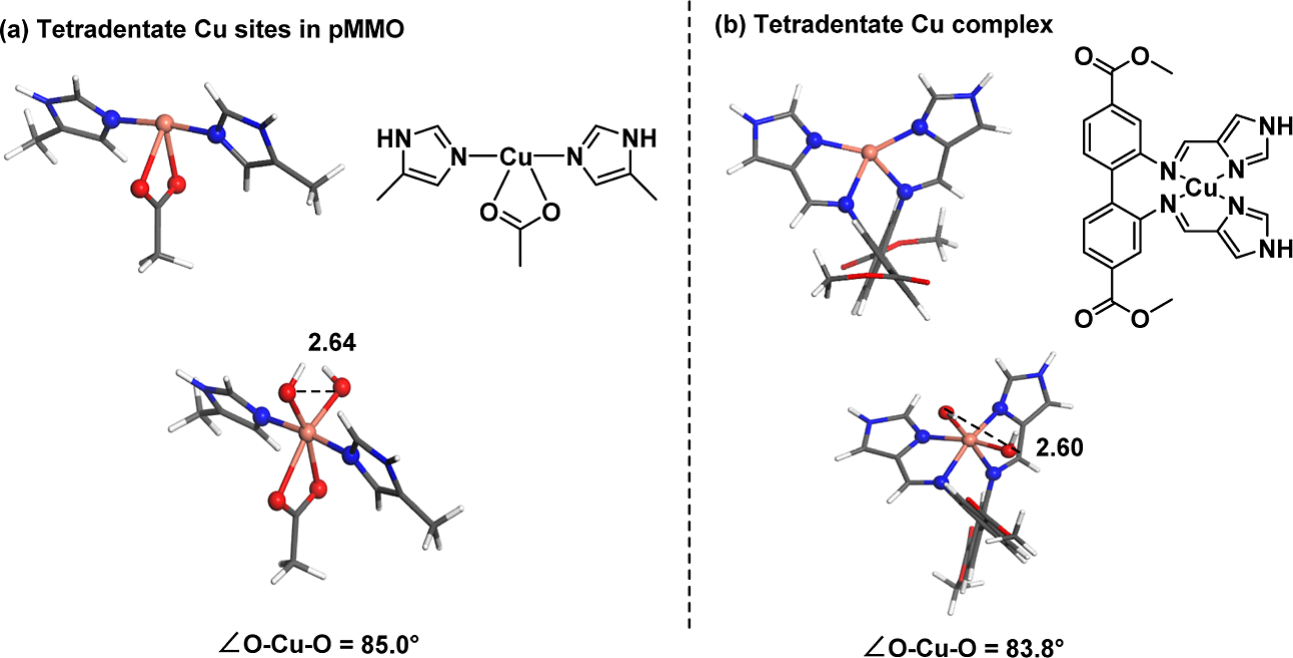
Cu(II)–(OH)_2_ geometries in tetracoordinate complexes after a CREST conformational search followed by geometry optimization: (a) the CuC site of pMMOs and (b) the N,N,N,N Cu complex recently synthesized in our group.

The tetradentate copper site in pMMO reported in Peng’s work was first considered, where copper is coordinated to two histidine ligands and an acetate ligand (Figure 13a). Since, in this study, we found that CREST is an effective approach for searching conformers, we took the Cu(II)–(OH)_2_ geometry from Peng’s work and performed a CREST conformational search. The results depicted in Figure 13a show that the dihydroxo species did not change to the *trans* position, the O–O bond distance and O–Cu–O angle remain the same (2.64 Å and 85.0°). This suggests that dihydroxo species are unlikely to change to the *trans* position on a hexacoordinated copper site, which originates from a tetracoordinated Cu complex. Furthermore, our group has previously reported a tetradentate Cu configuration (Figure 13b).^20^ To further verify our hypothesis, we repeated the conformational search using a Cu(II)–(OH)_2_ configuration in the hexacoordinated Cu(II) complex. As shown in Figure 13b, the dihydroxo species did not transition to a *trans* position either; the O–O bond distance and the O–Cu–O angle remained almost the same. The different reactivity observed between the highly unsaturated Cu–N,N,N system in the MOF and Cu-complexes with higher coordination modes may explain the higher activity for C–H bond activation reactions of the molecular Cu–N,N,N system with ester groups. As shown in Scheme 2, this system forms CuL_2_ complexes in solution, yielding Cu-atoms with six accessible N ligands, which may hinder the formation of *trans*-Cu(II)–(OH)_2_ species.

Upon comparing the tridentate complex with the Cu-MOF system and the tetradentate enzymatic and biomimetic systems, we can conclude that (a) when Cu(II)–(OH)_2_ is thermodynamically favorable, the hydroxo-ligands have a preference to adopt a *trans* configuration hindering the formation of Cu(II)–oxyl and opening intramolecular competing pathways in which the chelating ligand is oxidized; (b) the trans configuration is less preferred in the MOF, but still accessible and allowing for the intramolecular oxidation of the ligand; (c) high coordination numbers of Cu(II), i.e., four or more, can prevent the two hydroxo ligands adopting the *trans* configuration, facilitating the formation of the Cu(II)–oxyl species; (d) the *trans* configuration can also be avoided in the presence of water. This last observation gives insights into the formation of Cu(II)–oxyl species in LPMOs activated by H_2_O_2_, which feature similar tridentate Cu active sites. In these enzymes, the residues surrounding the active metal site can stabilize hydroxo species while preventing the formation of the *trans* configuration, thus favoring the generation of the Cu(II)–oxyl species.^45,46^ Moreover, we observed the rapid formation of Cu(II)–oxyl species from the intermediate Cu(II)–OH ··· OH and the geometric change in both Cu(I) and Cu(II) complexes when the simulated annealing method was applied. This highlights how simulated annealing AIMD simulations effectively explore the structural dynamics of these copper complexes, owing to the coordination flexibility of the copper center both in the Cu(I) and Cu(II) oxidation states.

## 4 Conclusion

In this study, we have unveiled new insights into the formation of Cu(II)–oxyl and Cu(II)–hydroxo species, showing how they are affected by different solvation and ligand environments. Our computational results highlight that Cu(II)–(OH)_2_, Cu(II)–OH···OH, and Cu(II)–OH···HO species can all participate in the homolytic cleavage of H_2_O_2_ by a bioinspired tridentate Cu(I) complex. Notably, Cu(II)–(OH)_2_ can easily isomerize into a *trans* configuration from which the formation of the Cu(II)–oxyl species becomes unlikely. However, the saturation of the metal coordination environment can effectively prevent the formation of this *trans*-bis-hydroxo complex. This knowledge can guide experimentalists in the further design of bioinspired copper complexes for catalytic C–H-oxidation reactions based on principles that can also be useful in the study of enzymatic systems.

## 5 Computational Methods

### 5.1 DFT Calculations

The static DFT calculations with cluster models were performed with the Gaussian16 software package.^47^ The structures were optimized using density functional theory (DFT) with the PBE0 functional,^48^ in conjunction with the def2-SVP basis set.^49^ Dispersion forces were taken into account using Grimme’s D3 model with Becke-Johnson damping.^50^ Vibrational frequencies were computed to verify that all stationary points represented energy minima (i.e., no imaginary frequencies). These calculations were also instrumental in determining thermochemical corrections (including zero-point, thermal, and entropy energies) referred to the standard state (*p* = 1 atm and *T* = 298.15 K). All energy values computed at the PBE0-D3(BJ)/def2-SVP level were further refined using the larger def2-TZVP basis set.^51^ The energies reported in the manuscript were obtained by adding the thermochemistry corrections to the refined potential energies. In addition, the Δ*G* of reactions involving a change in molecularity were corrected following the one-molar standard state correction (e.g., −1.9 kcal mol^–1^ for bimolecular reactions). The solvation effects of acetonitrile were modeled with the implicit SMD model for all calculations.^52^ The spin contamination was investigated and the results suggested that it was nearly inexistent in the triplet states and significant in the open-shell singlets (see Table S7).

For periodic models, all calculations were run in the CP2K 8.1 package^53^ using PBE functional^54^ and a mixed DZVP Gaussian and auxiliary plane-wave basis set.^55^ Grimme’s D3 dispersion model was employed to account for dispersion forces. The grid cutoffs were determined by the kinetic energy cutoff of the plane wave basis, set at 360 Ry, and the Brillouin zone was sampled at the gamma point. The original unit cell of UiO-67 was taken from the previous work in our group.^56^ A biphenyl linker in the original UiO-67 was removed and the functionalized ligand was installed and metaled with copper. Both the lattice constants and coordinates of the Cu–N,N,N UiO-67 MOF were optimized, resulting in *a* = 27.010 Å, *b* = 27.016 Å, *c* = 27.007 Å, and α = β = γ = 90°. For the calculations of intermediates, only coordinates were fully relaxed and optimized.

### 5.2 AIMD Simulations

Ab initio molecular dynamics (AIMD) simulations were performed in explicit acetonitrile solvent according to the Born–Oppenheimer approximation using the CP2K 8.1 program package.^53^ The initial configuration was created using the PACKMOL package,^57^ comprising the **T–1** (Cu(II)–(OH)_2_) intermediate surrounded by 85 acetonitrile molecules enclosed in a cubic box of 20.0 Å^3^, reproducing the density of this solvent (0.786 g/mL). Additionally, further AIMD simulations were performed for T-1 and T-12, incorporating the interactions of two water molecules with H_2_O_2_. The two water molecules were added manually to the simulation box containing 85 acetonitrile molecules. Periodic boundary conditions were applied in all AIMD calculations, which employed the PBE potential^54^ and a mixed basis set based on the Gaussian DZVP and the auxiliary plane-wave with a 250 Ry cutoff.^55^ Grimme’s D3 dispersion model was employed to account for dispersion forces.^50^ To explore the global minima on the potential surface, we used a simulated annealing approach at the beginning of the simulations.^37,38^ In simulated annealing, we start with an initial configuration at high temperature, which is slowly cooled down until it ceases to change significantly. The initial high temperature allows the system to overcome energy barriers, exploring larger areas of the potential energy surface.^58^ After the simulated annealing process, the system was equilibrated in the microcanonical (*NVE*) ensemble until an average temperature of 298 K was reached. The simulation was then extended in the canonical (*NVT*) ensemble, with a temperature of 298 K maintained with the CSVR thermostat.^59^ The trajectory was extended over 20 ps, employing a time step of 0.25 fs. The AIMD simulations of intermediates **T**–**5**, **T**_**MeCN**_**–1**, and **CSS**_**MeCN**_**–2** were performed with the same protocol under similar conditions.

### 5.3 Conformational Search in CREST

For automated conformational search, the Conformer-Rotamer Ensemble Sampling Tool (CREST) was employed.^60^ Calculations were conducted in CREST 2.11 at the GFN2-xTB level of theory,^39^ with an energy window of 6.0 kcal·mol^–1^. The structures used for the conformational search were initially preoptimized with the xTB method and subsequently searching conformers with CREST. The ALPB implicit solvation model was used for all calculations.^61^ The distinctive conformers generated by CREST were identified, and their single-point energies were computed at the level of PBE0/def2-TZVP in Gaussian 16. The conformers exhibiting the lowest DFT energy, or conformers with energy differences within 1.0 kcal·mol^–1^, were selected for further geometry optimization employing the methodology detailed in Section 5.1.

## Supporting Information Available

Additional energy profiles and AIMD trajectories (PDF)
Cartesian coordinates (XYZ)

## Supplementary Material

ic4c01948_si_001.pdf

ic4c01948_si_002.xyz

## References

[ref1] Vaaje-Kolstad G.; Westereng B.; Horn S. J.; Liu Z.; Zhai H.; Sørlie M.; Eijsink V. G. H. An Oxidative Enzyme Boosting the Enzymatic Conversion of Recalcitrant Polysaccharides. Science 2010, 330 (6001), 219–222. 10.1126/science.1192231. 20929773

[ref2] Munzone A.; Eijsink V. G. H.; Berrin J.-G.; Bissaro B. Expanding the catalytic landscape of metalloenzymes with lytic polysaccharide monooxygenases. Nat. Rev. Chem 2024, 8 (2), 106–119. 10.1038/s41570-023-00565-z. 38200220

[ref3] Bissaro B.; Eijsink V. G. H. Lytic polysaccharide monooxygenases: enzymes for controlled and site-specific Fenton-like chemistry. Essays Biochem. 2023, 67 (3), 575–584. 10.1042/ebc20220250. 36734231 PMC10154617

[ref4] Chylenski P.; Bissaro B.; Sørlie M.; Røhr Å. K.; Várnai A.; Horn S. J.; Eijsink V. G. H. Lytic Polysaccharide Monooxygenases in Enzymatic Processing of Lignocellulosic Biomass. ACS Catal. 2019, 9 (6), 4970–4991. 10.1021/acscatal.9b00246.

[ref5] Harris P. V.; Welner D.; McFarland K. C.; Re E.; Navarro Poulsen J.-C.; Brown K.; Salbo R.; Ding H.; Vlasenko E.; Merino S.; Xu F.; Cherry J.; Larsen S.; Lo Leggio L. Stimulation of Lignocellulosic Biomass Hydrolysis by Proteins of Glycoside Hydrolase Family 61: Structure and Function of a Large, Enigmatic Family. Biochemistry 2010, 49 (15), 3305–3316. 10.1021/bi100009p. 20230050

[ref6] Vu V. V.; Ngo S. T. Copper active site in polysaccharide monooxygenases. Coord. Chem. Rev. 2018, 368, 134–157. 10.1016/j.ccr.2018.04.005.

[ref7] Ciano L.; Davies G. J.; Tolman W. B.; Walton P. H. Bracing copper for the catalytic oxidation of C–H bonds. Nat. Catal. 2018, 1 (8), 571–577. 10.1038/s41929-018-0110-9.

[ref8] Quinlan R. J.; Sweeney M. D.; Lo Leggio L.; Otten H.; Poulsen J.-C. N.; Johansen K. S.; Krogh K. B. R. M.; Jørgensen C. I.; Tovborg M.; Anthonsen A.; Tryfona T.; Walter C. P.; Dupree P.; Xu F.; Davies G. J.; Walton P. H. Insights into the oxidative degradation of cellulose by a copper metalloenzyme that exploits biomass components. Proc. Natl. Acad. Sci. U.S.A. 2011, 108 (37), 15079–15084. 10.1073/pnas.1105776108. 21876164 PMC3174640

[ref9] Bissaro B.; Røhr Å. K.; Müller G.; Chylenski P.; Skaugen M.; Forsberg Z.; Horn S. J.; Vaaje-Kolstad G.; Eijsink V. G. H. Oxidative cleavage of polysaccharides by monocopper enzymes depends on H2O2. Nat. Chem. Biol. 2017, 13 (10), 1123–1128. 10.1038/nchembio.2470. 28846668

[ref10] Bissaro B.; Streit B.; Isaksen I.; Eijsink V. G. H.; Beckham G. T.; DuBois J. L.; Røhr Å. K. Molecular mechanism of the chitinolytic peroxygenase reaction. Proc. Natl. Acad. Sci. U.S.A. 2020, 117 (3), 1504–1513. 10.1073/pnas.1904889117. 31907317 PMC6983374

[ref11] Wang B.; Johnston E. M.; Li P.; Shaik S.; Davies G. J.; Walton P. H.; Rovira C. QM/MM Studies into the H2O2-Dependent Activity of Lytic Polysaccharide Monooxygenases: Evidence for the Formation of a Caged Hydroxyl Radical Intermediate. ACS Catal. 2018, 8 (2), 1346–1351. 10.1021/acscatal.7b03888.

[ref12] Hedegård E. D.; Ryde U. Molecular mechanism of lytic polysaccharide monooxygenases. Chem. Sci. 2018, 9 (15), 3866–3880. 10.1039/C8SC00426A. 29780519 PMC5935029

[ref13] Hedegård E. D.; Ryde U. Targeting the reactive intermediate in polysaccharide monooxygenases. J. Biol. Inorg. Chem. 2017, 22 (7), 1029–1037. 10.1007/s00775-017-1480-1. 28698982 PMC5613103

[ref14] Bertini L.; Breglia R.; Lambrughi M.; Fantucci P.; De Gioia L.; Borsari M.; Sola M.; Bortolotti C. A.; Bruschi M. Catalytic Mechanism of Fungal Lytic Polysaccharide Monooxygenases Investigated by First-Principles Calculations. Inorg. Chem. 2018, 57 (1), 86–97. 10.1021/acs.inorgchem.7b02005. 29232119

[ref15] Kim S.; Ståhlberg J.; Sandgren M.; Paton R. S.; Beckham G. T. Quantum mechanical calculations suggest that lytic polysaccharide monooxygenases use a copper-oxyl, oxygen-rebound mechanism. Proc. Natl. Acad. Sci. U.S.A. 2014, 111 (1), 149–154. 10.1073/pnas.1316609111. 24344312 PMC3890868

[ref16] Kim B.; Brueggemeyer M. T.; Transue W. J.; Park Y.; Cho J.; Siegler M. A.; Solomon E. I.; Karlin K. D. Fenton-like Chemistry by a Copper(I) Complex and H2O2 Relevant to Enzyme Peroxygenase C–H Hydroxylation. J. Am. Chem. Soc. 2023, 145 (21), 11735–11744. 10.1021/jacs.3c02273. 37195014 PMC10364799

[ref17] Kulkarni A. R.; Zhao Z.-J.; Siahrostami S.; Nørskov J. K.; Studt F. Monocopper Active Site for Partial Methane Oxidation in Cu-Exchanged 8MR Zeolites. ACS Catal. 2016, 6 (10), 6531–6536. 10.1021/acscatal.6b01895.

[ref18] Narsimhan K.; Iyoki K.; Dinh K.; Román-Leshkov Y. Catalytic Oxidation of Methane into Methanol over Copper-Exchanged Zeolites with Oxygen at Low Temperature. ACS Cent. Sci. 2016, 2 (6), 424–429. 10.1021/acscentsci.6b00139. 27413787 PMC4919767

[ref19] Kim S.; Ginsbach J. W.; Lee J. Y.; Peterson R. L.; Liu J. J.; Siegler M. A.; Sarjeant A. A.; Solomon E. I.; Karlin K. D. Amine Oxidative N-Dealkylation via Cupric Hydroperoxide Cu-OOH Homolytic Cleavage Followed by Site-Specific Fenton Chemistry. J. Am. Chem. Soc. 2015, 137 (8), 2867–2874. 10.1021/ja508371q. 25706825 PMC4482616

[ref20] Davydov R.; Herzog A. E.; Jodts R. J.; Karlin K. D.; Hoffman B. M. End-On Copper(I) Superoxo and Cu(II) Peroxo and Hydroperoxo Complexes Generated by Cryoreduction/Annealing and Characterized by EPR/ENDOR Spectroscopy. J. Am. Chem. Soc. 2022, 144 (1), 377–389. 10.1021/jacs.1c10252. 34981938 PMC8785356

[ref21] Maiti D.; Lee D.-H.; Gaoutchenova K.; Würtele C.; Holthausen M. C.; Narducci Sarjeant A.; Sundermeyer J.; Schindler S.; Karlin K. D. Reactions of a Copper(II) Superoxo Complex Lead to C-H and O-H Substrate Oxygenation: Modeling Copper-Monooxygenase C-H Hydroxylation. Angew. Chem., Int. Ed. 2008, 47 (1), 82–85. 10.1002/anie.200704389. 18022887

[ref22] Dietl N.; van der Linde C.; Schlangen M.; Beyer M. K.; Schwarz H. Diatomic [CuO]+ and Its Role in the Spin-Selective Hydrogen- and Oxygen-Atom Transfers in the Thermal Activation of Methane. Angew. Chem., Int. Ed. 2011, 50 (21), 4966–4969. 10.1002/anie.201100606. 21520367

[ref23] Dietl N.; Schlangen M.; Schwarz H. Thermal Hydrogen-Atom Transfer from Methane: The Role of Radicals and Spin States in Oxo-Cluster Chemistry. Angew. Chem., Int. Ed. 2012, 51 (23), 5544–5555. 10.1002/anie.201108363. 22431300

[ref24] Gagnon N.; Tolman W. B. [CuO]+ and [CuOH]2+ Complexes: Intermediates in Oxidation Catalysis?. Acc. Chem. Res. 2015, 48 (7), 2126–2131. 10.1021/acs.accounts.5b00169. 26075312 PMC4856291

[ref25] Gerz I.; Jannuzzi S. A. V.; Hylland K. T.; Negri C.; Wragg D. S.; Øien-Ødegaard S.; Tilset M.; Olsbye U.; DeBeer S.; Amedjkouh M. Structural Elucidation, Aggregation, and Dynamic Behaviour of N,N,N,N-Copper(I) Schiff Base Complexes in Solid and in Solution: A Combined NMR, X-ray Spectroscopic and Crystallographic Investigation. Eur. J. Inorg. Chem. 2021, 2021 (46), 4762–4775. 10.1002/ejic.202100722. 35874966 PMC9298233

[ref26] Concia A. L.; Beccia M. R.; Orio M.; Ferre F. T.; Scarpellini M.; Biaso F.; Guigliarelli B.; Réglier M.; Simaan A. J. Copper Complexes as Bioinspired Models for Lytic Polysaccharide Monooxygenases. Inorg. Chem. 2017, 56 (3), 1023–1026. 10.1021/acs.inorgchem.6b02165. 28060494

[ref27] Bete S. C.; Würtele C.; Otte M. A bio-inspired imidazole-functionalised copper cage complex. Chem. Commun. 2019, 55 (30), 4427–4430. 10.1039/C9CC00437H. 30916684

[ref28] Donoghue P. J.; Gupta A. K.; Boyce D. W.; Cramer C. J.; Tolman W. B. An Anionic, Tetragonal Copper(II) Superoxide Complex. J. Am. Chem. Soc. 2010, 132 (45), 15869–15871. 10.1021/ja106244k. 20977226 PMC3013377

[ref29] Ramírez E.; Hossain M. K.; Flores-Alamo M.; Haukka M.; Nordlander E.; Castillo I. Oxygen Transfer from Trimethylamine N-Oxide to CuI Complexes Supported by Pentanitrogen Ligands. Eur. J. Inorg. Chem. 2020, 2020 (29), 2798–2808. 10.1002/ejic.202000488.

[ref30] Baek J.; Rungtaweevoranit B.; Pei X.; Park M.; Fakra S. C.; Liu Y.-S.; Matheu R.; Alshmimri S. A.; Alshehri S.; Trickett C. A.; Somorjai G. A.; Yaghi O. M. Bioinspired Metal–Organic Framework Catalysts for Selective Methane Oxidation to Methanol. J. Am. Chem. Soc. 2018, 140 (51), 18208–18216. 10.1021/jacs.8b11525. 30525562

[ref31] Gerz I.; Aunan E. S.; Finelli V.; Abu Rasheed M.; Deplano G.; Cortez S P R.; Schmidtke I. L.; Wragg D. S.; Signorile M.; Hylland K. T.; Borfecchia E.; Lillerud K. P.; Bordiga S.; Olsbye U.; Amedjkouh M. Enabling a bioinspired N,N,N-copper coordination motif through spatial control in UiO-67: synthesis and reactivity. Dalton Trans. 2024, 53, 8141–8153. 10.1039/d3dt03096b. 38483202 PMC11091859

[ref32] Kjaergaard C. H.; Qayyum M. F.; Wong S. D.; Xu F.; Hemsworth G. R.; Walton D. J.; Young N. A.; Davies G. J.; Walton P. H.; Johansen K. S.; Hodgson K. O.; Hedman B.; Solomon E. I. Spectroscopic and computational insight into the activation of O2 by the mononuclear Cu center in polysaccharide monooxygenases. Proc. Natl. Acad. Sci. U.S.A. 2014, 111 (24), 8797–8802. 10.1073/pnas.1408115111. 24889637 PMC4066490

[ref33] Wang B.; Walton P. H.; Rovira C. Molecular Mechanisms of Oxygen Activation and Hydrogen Peroxide Formation in Lytic Polysaccharide Monooxygenases. ACS Catal. 2019, 9 (6), 4958–4969. 10.1021/acscatal.9b00778. 32051771 PMC7007194

[ref34] Lee I.; Lee M.-S.; Tao L.; Ikuno T.; Khare R.; Jentys A.; Huthwelker T.; Borca C. N.; Kalinko A.; Gutiérrez O. Y.; Govind N.; Fulton J. L.; Hu J. Z.; Glezakou V.-A.; Rousseau R.; Sanchez-Sanchez M.; Lercher J. A. Activity of Cu–Al–Oxo Extra-Framework Clusters for Selective Methane Oxidation on Cu-Exchanged Zeolites. JACS Au 2021, 1 (9), 1412–1421. 10.1021/jacsau.1c00196. 34604851 PMC8479761

[ref35] Xu R.; Liu N.; Dai C.; Li Y.; Zhang J.; Wu B.; Yu G.; Chen B. H2O-Built Proton Transfer Bridge Enhances Continuous Methane Oxidation to Methanol over Cu-BEA Zeolite. Angew. Chem., Int. Ed. 2021, 60 (30), 16634–16640. 10.1002/anie.202105167. 33982395

[ref36] Peng W.; Qu X.; Shaik S.; Wang B. Deciphering the oxygen activation mechanism at the CuC site of particulate methane monooxygenase. Nat. Catal. 2021, 4 (4), 266–273. 10.1038/s41929-021-00591-4.

[ref37] Kirkpatrick S.; Gelatt C. D.; Vecchi M. P. Optimization by Simulated Annealing. Science 1983, 220 (4598), 671–680. 10.1126/science.220.4598.671. 17813860

[ref38] Hartke B.; Carter E. A. Ab initio molecular dynamics with correlated molecular wave functions: Generalized valence bond molecular dynamics and simulated annealing. J. Chem. Phys. 1992, 97 (9), 6569–6578. 10.1063/1.463660.

[ref39] Bannwarth C.; Ehlert S.; Grimme S. GFN2-xTB—An Accurate and Broadly Parametrized Self-Consistent Tight-Binding Quantum Chemical Method with Multipole Electrostatics and Density-Dependent Dispersion Contributions. J. Chem. Theory Comput. 2019, 15 (3), 1652–1671. 10.1021/acs.jctc.8b01176. 30741547

[ref40] Chen J.; Unjaroen D.; Stepanovic S.; van Dam A.; Gruden M.; Browne W. R. Selective Photo-Induced Oxidation with O2 of a Non-Heme Iron(III) Complex to a Bis(imine-pyridyl)iron(II) Complex. Inorg. Chem. 2018, 57 (8), 4510–4515. 10.1021/acs.inorgchem.8b00187. 29601196 PMC5906753

[ref41] Liu S.-C.; Zhu X.-R.; Liu D.-Y.; Fang D.-C. DFT calculations in solution systems: solvation energy, dispersion energy and entropy. Phys. Chem. Chem. Phys. 2023, 25 (2), 913–931. 10.1039/D2CP04720A. 36519338

[ref42] Plata R. E.; Singleton D. A. A Case Study of the Mechanism of Alcohol-Mediated Morita Baylis–Hillman Reactions. The Importance of Experimental Observations. J. Am. Chem. Soc. 2015, 137 (11), 3811–3826. 10.1021/ja5111392. 25714789 PMC4379969

[ref43] Mammen M.; Shakhnovich E. I.; Deutch J. M.; Whitesides G. M. Estimating the Entropic Cost of Self-Assembly of Multiparticle Hydrogen-Bonded Aggregates Based on the Cyanuric Acid·Melamine Lattice. J. Org. Chem. 1998, 63 (12), 3821–3830. 10.1021/jo970944f.

[ref44] Singh R.; Ganguly G.; Malinkin S. O.; Demeshko S.; Meyer F.; Nordlander E.; Paine T. K. A Mononuclear Nonheme Iron(IV)-Oxo Complex of a Substituted N4Py Ligand: Effect of Ligand Field on Oxygen Atom Transfer and C–H Bond Cleavage Reactivity. Inorg. Chem. 2019, 58 (3), 1862–1876. 10.1021/acs.inorgchem.8b02577. 30644733

[ref45] Schröder G. C.; O’Dell W. B.; Webb S. P.; Agarwal P. K.; Meilleur F. Capture of activated dioxygen intermediates at the copper-active site of a lytic polysaccharide monooxygenase. Chem. Sci. 2022, 13 (45), 13303–13320. 10.1039/D2SC05031E. 36507176 PMC9683017

[ref46] Hall K. R.; Joseph C.; Ayuso-Fernández I.; Tamhankar A.; Rieder L.; Skaali R.; Golten O.; Neese F.; Røhr Å. K.; Jannuzzi S. A. V.; DeBeer S.; Eijsink V. G. H.; Sørlie M. A Conserved Second Sphere Residue Tunes Copper Site Reactivity in Lytic Polysaccharide Monooxygenases. J. Am. Chem. Soc. 2023, 145 (34), 18888–18903. 10.1021/jacs.3c05342. 37584157 PMC10472438

[ref47] Frisch M. J.; Trucks G. W.; Schlegel H. B.; Scuseria G. E.; Robb M. A.; Cheeseman J. R.; Scalmani G.; Barone V.; Petersson G. A.; Nakatsuji H.; Li X.; Caricato M.; Marenich A. V.; Bloino J.; Janesko B. G.; Gomperts R.; Mennucci B.; Hratchian H. P.; Ortiz J. V.; Izmaylov A. F.; Sonnenberg J. L.; Williams; Ding F.; Lipparini F.; Egidi F.; Goings J.; Peng B.; Petrone A.; Henderson T.; Ranasinghe D.; Zakrzewski V. G.; Gao J.; Rega N.; Zheng G.; Liang W.; Hada M.; Ehara M.; Toyota K.; Fukuda R.; Hasegawa J.; Ishida M.; Nakajima T.; Honda Y.; Kitao O.; Nakai H.; Vreven T.; Throssell K.; Montgomery J. A. Jr.; Peralta J. E.; Ogliaro F.; Bearpark M. J.; Heyd J. J.; Brothers E. N.; Kudin K. N.; Staroverov V. N.; Keith T. A.; Kobayashi R.; Normand J.; Raghavachari K.; Rendell A. P.; Burant J. C.; Iyengar S. S.; Tomasi J.; Cossi M.; Millam J. M.; Klene M.; Adamo C.; Cammi R.; Ochterski J. W.; Martin R. L.; Morokuma K.; Farkas O.; Foresman J. B.; Fox D. J. Gaussian 16. Rev. C.01; Gaussian, Inc.: Wallingford, CT, 2016.

[ref48] Adamo C.; Barone V. Toward reliable density functional methods without adjustable parameters: The PBE0 model. J. Chem. Phys. 1999, 110 (13), 6158–6170. 10.1063/1.478522.

[ref49] Weigend F.; Ahlrichs R. Balanced basis sets of split valence, triple zeta valence and quadruple zeta valence quality for H to Rn: Design and assessment of accuracy. Phys. Chem. Chem. Phys. 2005, 7 (18), 3297–3305. 10.1039/b508541a. 16240044

[ref50] Grimme S.; Antony J.; Ehrlich S.; Krieg H. A consistent and accurate ab initio parametrization of density functional dispersion correction (DFT-D) for the 94 elements H-Pu. J. Chem. Phys. 2010, 132 (15), 154104 10.1063/1.3382344. 20423165

[ref51] Weigend F. Hartree–Fock exchange fitting basis sets for H to Rn. J. Comput. Chem. 2008, 29 (2), 167–175. 10.1002/jcc.20702. 17568435

[ref52] Marenich A. V.; Cramer C. J.; Truhlar D. G. Universal Solvation Model Based on Solute Electron Density and on a Continuum Model of the Solvent Defined by the Bulk Dielectric Constant and Atomic Surface Tensions. J. Phys. Chem. B 2009, 113 (18), 6378–6396. 10.1021/jp810292n. 19366259

[ref53] Kühne T. D.; Iannuzzi M.; Del Ben M.; Rybkin V. V.; Seewald P.; Stein F.; Laino T.; Khaliullin R. Z.; Schütt O.; Schiffmann F.; Golze D.; Wilhelm J.; Chulkov S.; Bani-Hashemian M. H.; Weber V.; Borštnik U.; Taillefumier M.; Jakobovits A. S.; Lazzaro A.; Pabst H.; Müller T.; Schade R.; Guidon M.; Andermatt S.; Holmberg N.; Schenter G. K.; Hehn A.; Bussy A.; Belleflamme F.; Tabacchi G.; Glöß A.; Lass M.; Bethune I.; Mundy C. J.; Plessl C.; Watkins M.; VandeVondele J.; Krack M.; Hutter J. CP2K: An electronic structure and molecular dynamics software package - Quickstep: Efficient and accurate electronic structure calculations. J. Chem. Phys. 2020, 152 (19), 194103 10.1063/5.0007045. 33687235

[ref54] Perdew J. P.; Burke K.; Ernzerhof M. Generalized Gradient Approximation Made Simple. Phys. Rev. Lett. 1996, 77 (18), 3865–3868. 10.1103/PhysRevLett.77.3865. 10062328

[ref55] Godbout N.; Salahub D. R.; Andzelm J.; Wimmer E. Optimization of Gaussian-type basis sets for local spin density functional calculations. Part I. Boron through neon, optimization technique and validation. Optim. Tech. Validation 1992, 70 (2), 560–571. 10.1139/v92-079.

[ref56] Gutterød E. S.; Lazzarini A.; Fjermestad T.; Kaur G.; Manzoli M.; Bordiga S.; Svelle S.; Lillerud K. P.; Skúlason E.; Øien-Ødegaard S.; Nova A.; Olsbye U. Hydrogenation of CO2 to Methanol by Pt Nanoparticles Encapsulated in UiO-67: Deciphering the Role of the Metal–Organic Framework. J. Am. Chem. Soc. 2020, 142 (2), 999–1009. 10.1021/jacs.9b10873. 31794194

[ref57] Martínez L.; Andrade R.; Birgin E. G.; Martínez J. M. PACKMOL: A package for building initial configurations for molecular dynamics simulations. J. Comput. Chem. 2009, 30 (13), 2157–2164. 10.1002/jcc.21224. 19229944

[ref58] Mundim K. C.; Tsallis C. Geometry optimization and conformational analysis through generalized simulated annealing. Int. J. Quantum Chem. 1998, 58 (4), 373–381. 10.1002/(SICI)1097-461X(1996)58:4<373::AID-QUA6>3.0.CO;2-V.

[ref59] Bussi G.; Donadio D.; Parrinello M. Canonical sampling through velocity rescaling. J. Chem. Phys. 2007, 126 (1), 014101 10.1063/1.2408420. 17212484

[ref60] Pracht P.; Bohle F.; Grimme S. Automated exploration of the low-energy chemical space with fast quantum chemical methods. Phys. Chem. Chem. Phys. 2020, 22 (14), 7169–7192. 10.1039/C9CP06869D. 32073075

[ref61] Ehlert S.; Stahn M.; Spicher S.; Grimme S. Robust and Efficient Implicit Solvation Model for Fast Semiempirical Methods. J. Chem. Theory Comput. 2021, 17 (7), 4250–4261. 10.1021/acs.jctc.1c00471. 34185531

