# Supplementary material for: Copper(II)-Oxyl Formation in a Biomimetic Complex Activated by Hydrogen Peroxide: The Key Role of Trans-Bis(Hydroxo) Species
Source: Inorg Chem. 2024 Nov 25;63(49):23082–94. doi: 10.1021/acs.inorgchem.4c01948 (PMC11632775; doi:10.1021/acs.inorgchem.4c01948)
Supplement: Supplementary file 1 — ic4c01948_si_001.pdf [file ic4c01948_si_001.pdf]

Supporting Information for:

Copper(II)-Oxyl Formation in a Biomimetic Complex Activated by Hydrogen Peroxide: The Key Role of Trans-Bis(Hydroxo) Species

Ning Cao,[a,b] Abril C. Castro,[b,] David Balcells,[b] Unni Olsbye,[a] and Ainara Nova[a,b,*]*

[a] Department of Chemistry, Centre for Materials and Nanoscience (SMN), University of Oslo, P.O. Box 1033, Blindern, NO-0315 Oslo, Norway
[b] Hylleraas Centre for Quantum Molecular Sciences, Department of Chemistry, University of Oslo, P. O. Box 1033, Blindern, N-0315, Oslo, Norway.

corresponding authors' email address:
a.n.flores@kjemi.uio.no
a.c.aguilera@kjemi.uio.no

Contents

Figure S1. The constrained cluster model from the Cu UiO-67 MOF. S4

Figure S2. The energy profiles of the Cu(I) complex with ester groups activated by H_2O_2. S5

Figure S3. The relaxed scan of the O–O bond of H_2O_2 in open-shell singlet and triplet states. S6

Figure S4. The relaxed scan of the Cu–O bond. S6

Figure S5. The evolution of bond distances in **T–1** intermediate along the NVT trajectory.S7

Table S1. Comparison of selected bond distances in the optimized **T–1** intermediate and the geometry obtained by AIMD, including the mean, minimum, and maximum values along the NVT trajectory. S7

Figure S6. Conformational search of **T–1** intermediate in CREST. S8

Figure S7. The evolution of **T–2** intermediate along the NVE trajectory. S8

Figure S8. Comparison among the **T–4**, **T–6**, **T–7**, and intermediates in their closed-shell singlet states. S9

Figure S9. Changes in the spin populations on Cu, N_{amine}, and two O atoms from hydroxo groups with the increase in the O–O bond distance. S10

Table S2. The evolution of NPA charges on Cu, OH, and N amine. S10

Figure S10. The evolution of bond distances in **T–5** intermediate along the NVT trajectory. S11

Table S3. Comparison of selected bond distances in the optimized **T–5** intermediate and the geometry obtained by AIMD, including the mean, minimum, and maximum values along the NVT trajectory. S11

Figure S11. Comparison between the results from AIMD simulations and conformational search with CREST. S12

Figure S12. The energy profile of a hydroxo ligand abstracting H from the ligand without the assistance of water. S13

Figure S13. The coordination of a water molecule to the Cu(I) complex. S13

Figure S14. The evolution of bond distances in **T–1** intermediate with two water molecules along the NVT trajectory. S14

Table S4. Comparison of selected bond distances in the optimized **T–1** intermediate with two water molecules and the geometry obtained by AIMD, including the mean, minimum, and maximum values along the NVT trajectory. S14

Figure S15. Geometry Optimization on Cu(II)–$(OH)_2$ with two water molecules S15

Figure S16. Changes in the spin populations on Cu, N_{amine}, and two O atoms from hydroxo groups with the increase in the O–O bond distance when water is present. S15

Figure S17. The evolution of **T–12** intermediate along the NVE trajectory. S15

Figure S18. The evolution of bond distances in $\mathbf{T_{MeCN}}$–**1** intermediate along the NVT trajectory. S16
Table S5. Comparison of selected bond distances in the optimized $\mathbf{T_{MeCN}}$–**1** intermediate and the geometry obtained by AIMD, including the mean, minimum, and maximum values along the NVT trajectory. S16
Figure S19. The evolution of bond distances in $\mathbf{CSS_{MeCN}}$–**2** intermediate along the NVT trajectory. S17
Table S6. Comparison of selected bond distances in the optimized $\mathbf{CSS_{MeCN}}$–**2** intermediate and the geometry obtained by AIMD, including the mean, minimum, and maximum values along the NVT trajectory. S17
Table S7. The <S^2> values of intermediates and transition states shown in the energy profiles. S18

The constrained cluster model

The extracted Cu complex from the optimized Cu MOF is depicted in Figure S2a. To maintain the rigidity of the MOF structure, we constructed a cluster model where sodium atoms replaced the Zr-nodes to balance charges, and the oxygen atoms of the carboxylates were frozen to their positions in the MOF. The constrained model was then optimized in Gaussian (see Figure S2b). Compared to the free cluster model, the constrained cluster model is less distorted, with the angle of N_{py}–Cu–N_{IM} being 155.6°. Although the bond distances of Cu–Npy, Cu–N_{IM}, and Cu–N_{amine} in the constrained model are longer than those in the MOF structure, the differences are within 0.1 Å.

The coordination of H_2O_2 to Cu in the constrained model with carboxylate groups (**CSS$_{ONa}$–1**) is 11.9 kcal mol-1, whereas it is –2.1 kcal mol^{-1} in the free model without carboxylate groups (**CSS–1**). To evaluate whether the origin of the energy difference was the carboxylate groups or the geometry constraints, the coordination of H_2O_2 to Cu was also calculated with the same model without geometry constraints (**CSS$_{ONa}$–2'**). The similar energies obtained for the two models indicate that the higher energy barrier obtained with the carboxylate systems is due to the carboxylate groups, which hinders the H_2O_2 coordination to Cu.

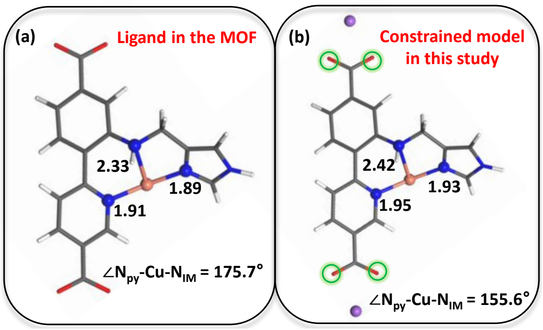

Figure S1 The extracted copper metallated ligand from UiO-67 MOF as shown in Figure 1. (**b**) The constrained cluster model used for comparisons in this study. Sodium atoms were added to balance the charge. The constrained atoms were highlighted in green circles. Black labels highlight bond distances (angstrom, Å) in each complex.

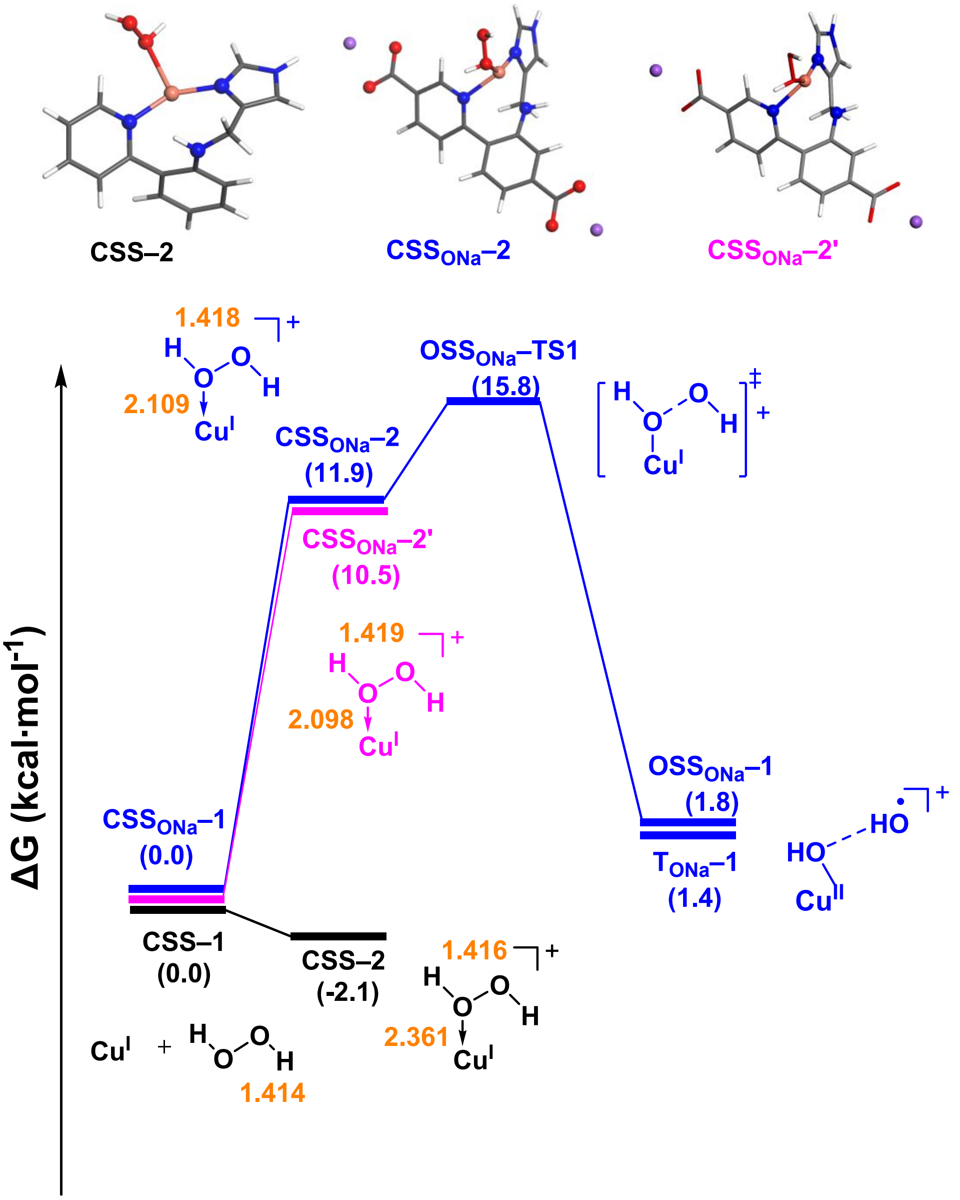

Figure S2 Gibbs free energy profile for the coordination of H_2O_2 to Cu in the cluster without carboxylate groups (**CSS-1**), with carboxylate groups (**CSS$_{ONa}$-1'**) and with constrained carboxylate groups (**CSS$_{ONa}$-1**).

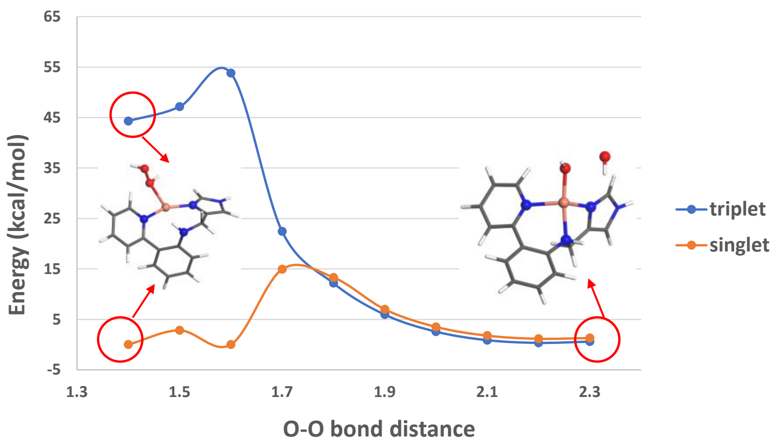

Figure S3 The relaxed scan of the O–O bond of H_2O_2 in open-shell singlet and triplet states, starting from the intermediate **CSS–2**. In the singlet states, the first three constrained calculations are in closed-shell singlets, while others are in open-shell singlets.

Figure S3 shows our attempt to determine the O-O activation pathway by a singlet to triplet spin crossover pathway, which showed an energy barrier of 15.9 kcal mol^{-1}, which is higher than 11.8 kcal mol^{-1} through OSS–TS1. The relaxed scan displayed another reaction pathway that connects CSS–2 and T–2, but with higher energy and thus is disregarded. To prove the connection between **CSS–2** and **OSS–1** via **OSS–TS1**, we ran an IRC calculation. Unfortunately, the calculation stopped in the direction leading to **OSS–1**. Therefore, we displaced the geometry exactly along the vibrational mode, corresponding to an elongation of the O–O bond from 1.69 Å (in the TS) to 1.76 Å, followed by a full geometry optimization. This calculation led to **OSS–1** instead of an open-shell single species with an OH group loosely coordinated as in **T–2**.

Additionally, a relaxed scan of the Cu–O bond, starting from intermediate **T–1**, revealed a saddle point, though the corresponding transition state could not be correctly located. However, we estimate the energy barrier between **T–2** and **T–1** to be only 0.3 kcal/mol, which is negligible.

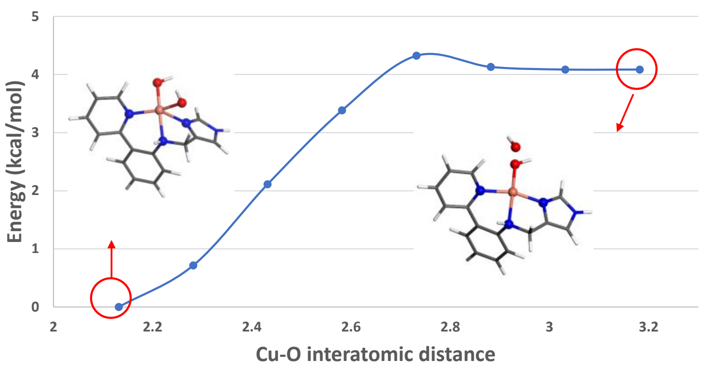

Figure S4 The relaxed scan of the Cu–O bond, starting from the intermediate **T–1**.

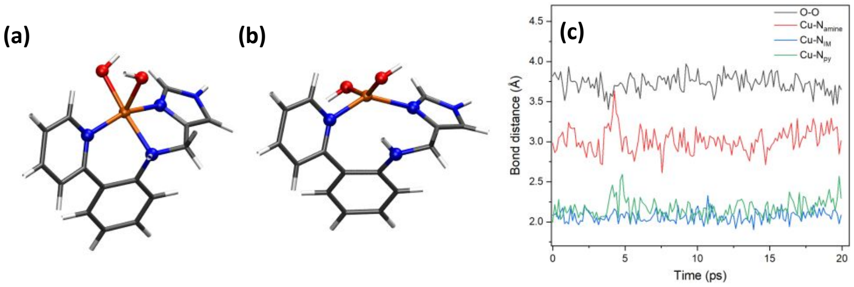

Figure S5. (a) The optimized **T–1** intermediate in Gaussian 16; (b) The abstracted geometry from CP2K after the AIMD simulations; (c) The evolution of the Cu–N_{py}, Cu–N_{IM}, Cu–N_{amine}, O–O, Cu–O_a, and Cu–O_b bond distances in **T**–**1** intermediate along the NVT trajectory.

Table S1 Comparison of selected bond distances in the optimized **T–1** intermediate and the geometry obtained by AIMD, including the mean, minimum, and maximum values along the NVT trajectory.

	PBE0-D3/SVP, implicit MeCN solvent	PBE-D3/DZVP, explicit MeCN solvent		
		MEAN	MIN	MAX
Cu–N_{Py}	2.04	2.18	1.90	2.67
Cu–N_{IM}	2.01	2.07	1.87	2.38
Cu–N_{amine}	2.09	3.01	2.59	3.74
O–O	2.24	3.72	3.36	4.02
Cu–O_a	2.15	1.92	1.77	2.11
Cu–O_b	1.92	1.89	1.74	2.09

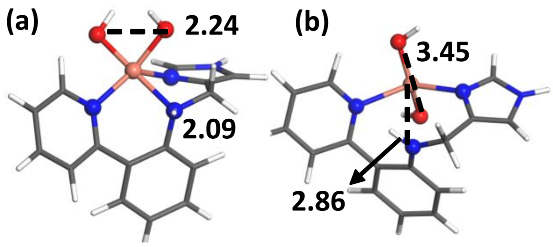

Figure S6 (a) Initial geometry of **T–1** optimized in Gaussian 16; (b) The geometry after searching conformers in CREST without optimization at the DFT level.

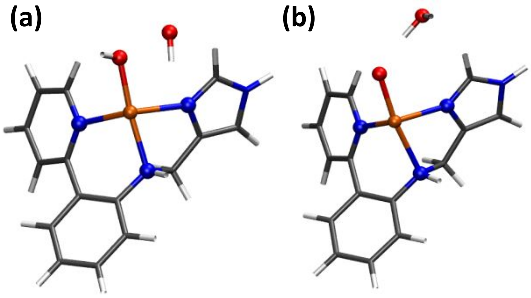

Figure S7 (a) Initial geometry of **T–2** optimized in Gaussian 16; (b) The abstracted geometry from CP2K after the AIMD simulations.

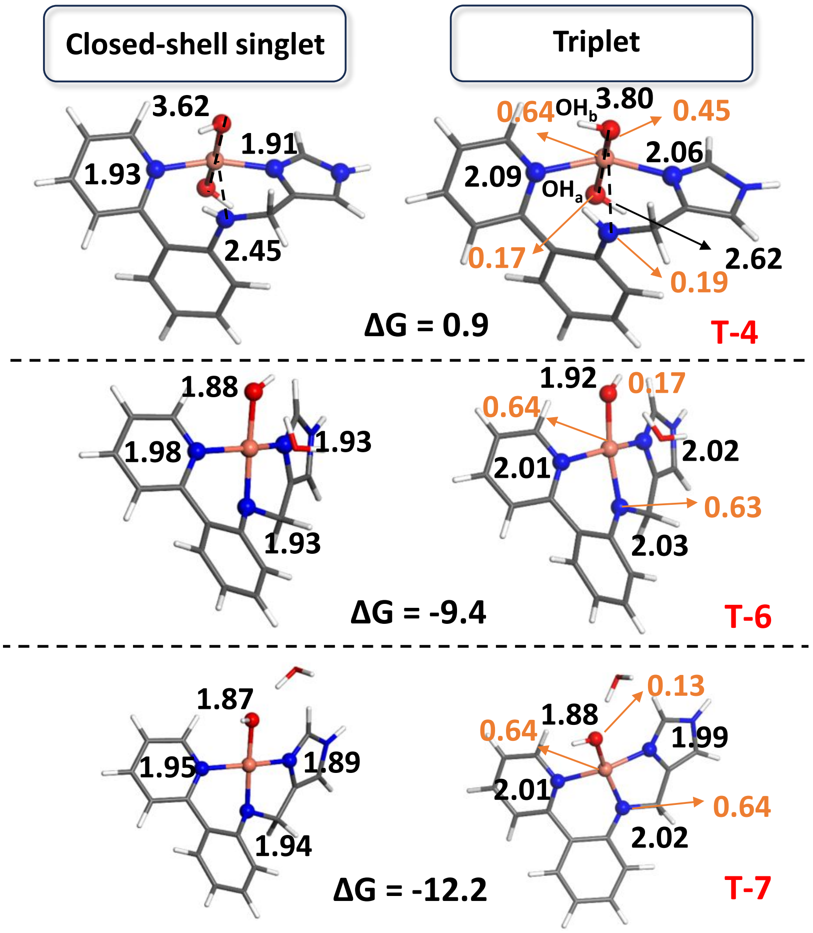

Figure S8 Comparison among the **T–4**, **T–6**, **T–7**, and intermediates in their closed-shell singlet states. The interatomic distances are labeled in black and the spin population is labeled in orange.

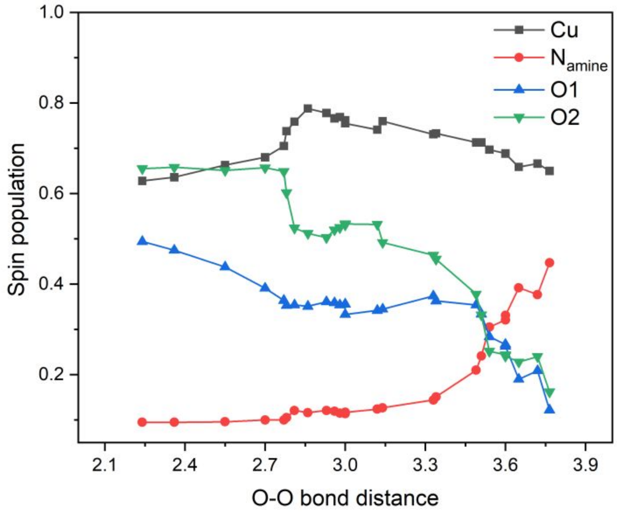

Figure S9 Changes in the spin populations on Cu, N_{amine}, and two O atoms from hydroxo groups with the increase in the O–O in Å.

Table S2 The evolution of NPA charges on Cu, OH, and N_{amine} along the energy profile.

Intermediate	Cu	O_a in OH_a	O_b in OH_b	N_{amine}
**T-1**	0.99	–0.81	–0.688	–0.70
**T-4**	1.36	–1.12	–0.902	–0.57
**T-5**	1.34	–1.08	/	–0.51
**T-7**	1.38	–1.14	/	–0.51
**T-8**	1.38	/	/	–0.71
**CSS-4**	1.43	–0.94	–0.96	–0.73
**CSS-5**	0.94	/	/	–0.60
**CSS-6**	0.72	/	/	–0.52

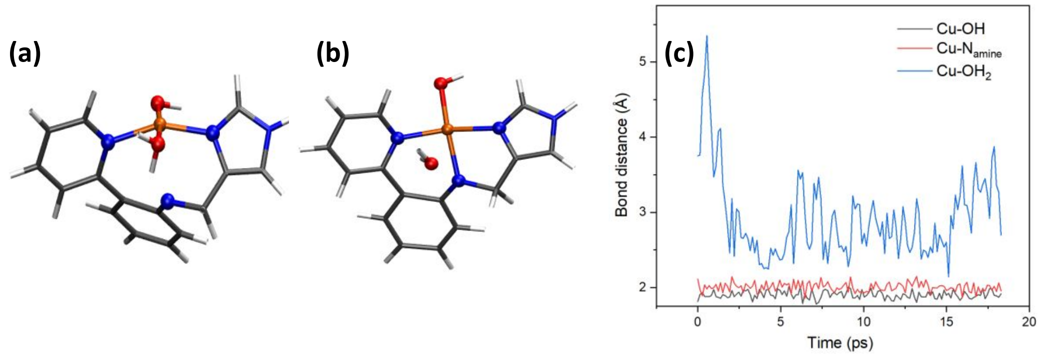

Figure S10 (a) The optimized **T–5** intermediate in Gaussian 16; (b) The abstracted geometry from CP2K after the AIMD simulations; (c) The evolution of the Cu–N_{py}, Cu–N_{IM}, Cu–N_{amine}, Cu–OH_2, and Cu–OH bond distances in **T–5** intermediate along the NVT trajectory.

Table S3 Comparison of selected bond distances in the optimized **T–5** intermediate and the geometry obtained by AIMD, including the mean, minimum, and maximum values along the NVT trajectory.

	PBE0-D3/SVP, implicit MeCN solvent	PBE-D3/DZVP, explicit MeCN solvent		
		MEAN	MIN	MAX
Cu–N_{Py}	2.05	2.05	1.82	2.29
Cu–N_{IM}	2.01	2.01	1.84	2.25
Cu–N_{amine}	2.48	2.01	1.83	2.21
Cu–OH_2	2.16	2.92	2.11	5.42
Cu–OH	1.86	1.90	1.78	2.10

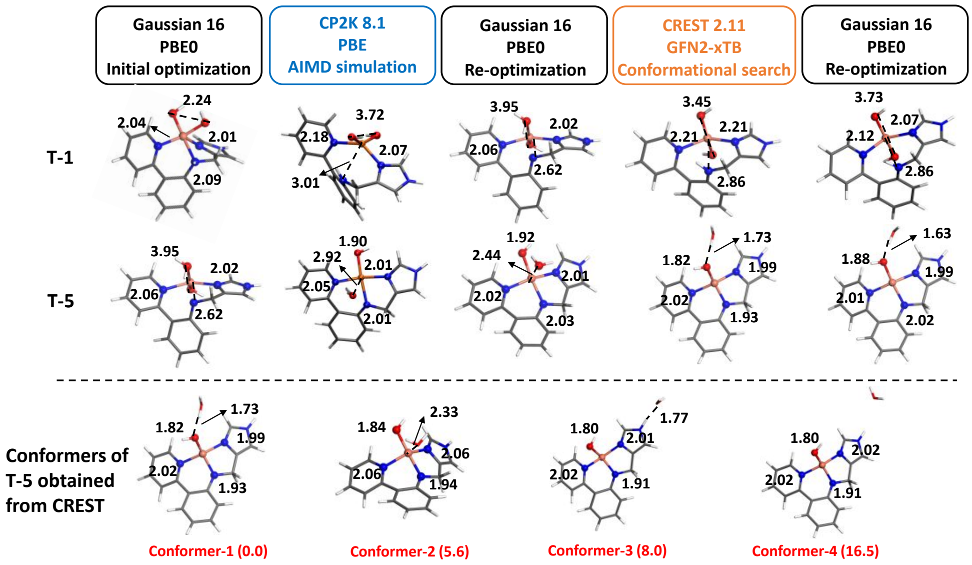

Figure S11 Comparison between the results from AIMD simulations and conformational search with CREST.

First column: Initial structure taken from Gaussian, optimized at the PBE0 level.

Second column: Final structure after AIMD simulation.

Third column: Extracted structure from CP2K, optimized in Gaussian at the PBE0 level.

Fourth column: Final structure after conformer search with CREST.

Fifth column: Extracted structure from CP2K, optimized in Gaussian at the PBE0 level.

The bond distances are labelled in black.

For intermediate **T–1**, CREST generated several identical conformers and successfully reproduced a structure very similar to that obtained from AIMD simulations. However, for intermediate **T–5**, CREST produced various conformers with the water molecule in different positions. We optimized these conformers using the PBE0 functional in Gaussian and selected the one with the lowest energy. In **Conformer–1**, water interacts with the hydroxyl group, resulting in the lowest energy configuration. In **Conformer–2**, water interacts with the copper as in the conformer obtained using AIMD simulations. The energy of this conformer is higher by 5.6 kcal mol^{-1} compared to **Conformer–1**. In **Conformer–3** and **–4,** water interacts with the ligand. These structures have energies of 8.0 and 16.5 kcal mol^{-1}, respectively, higher than **Conformer–1**.

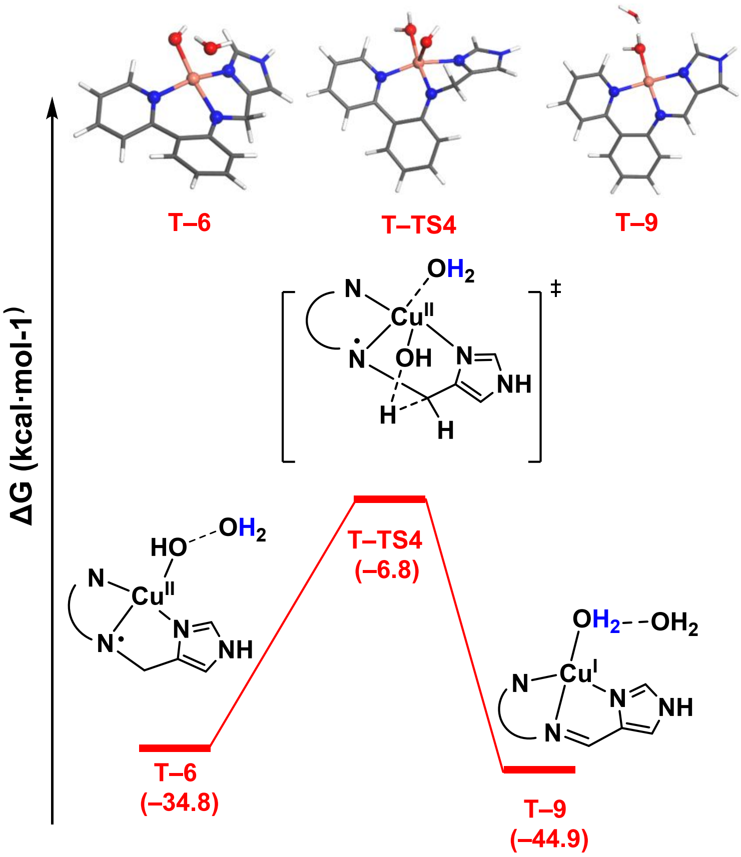

Figure S12 Starting from **T–6**, the energy profile of a hydroxo ligand abstracting H from the ligand without the assistance of water.

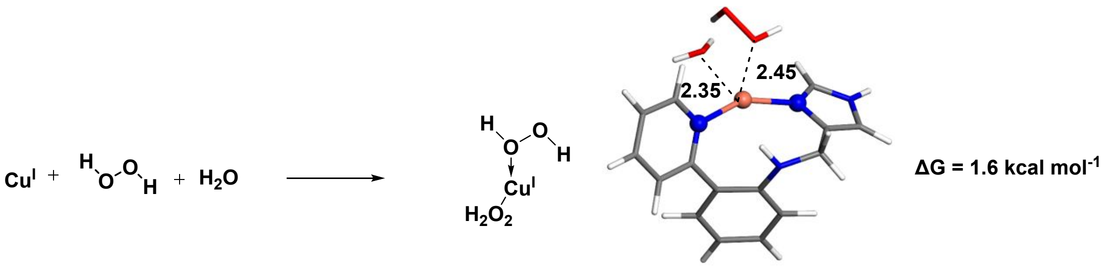

Figure S13 The coordination of a water molecule to the Cu(I) complex

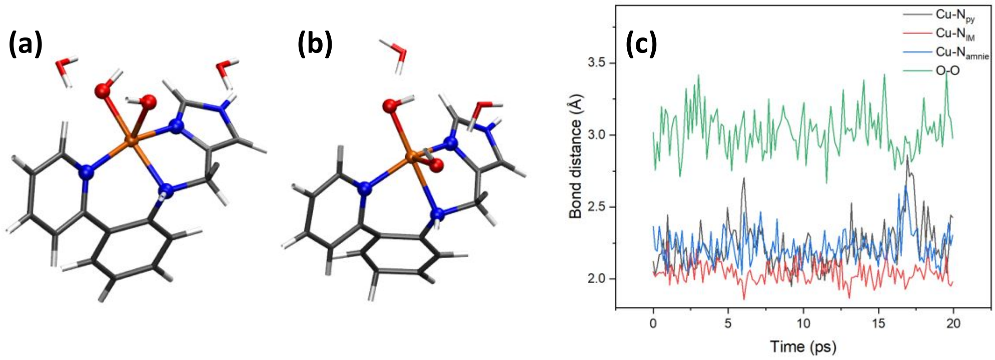

Figure S14 (a) The **T–1** intermediate with two water molecules without optimization; (b) The abstracted geometry from CP2K after the AIMD simulations; (c) The evolution of the Cu–N_{py}, Cu–N_{IM}, Cu–N_{amine}, O–O, Cu–O_a, and Cu–O_b bond distances in **T–1** intermediate with two water molecules along the NVT trajectory.

Table S4 Comparison of selected bond distances in the optimized **T–1** intermediate with two water molecules and the geometry obtained by AIMD, including the mean, minimum, and maximum values along the NVT trajectory.

	PBE0-D3/SVP, implicit MeCN solvent	PBE-D3/DZVP, explicit MeCN solvent		
		MEAN	MIN	MAX
Cu–N_{Py}	2.04	2.24	1.95	2.86
Cu–N_{IM}	2.01	2.04	1.86	2.30
Cu–N_{amine}	2.09	2.22	1.98	2.70
O–O	2.24	3.01	2.52	3.51
Cu–O_a	2.15	2.04	1.82	2.40
Cu–O_b	1.92	1.95	1.80	2.21

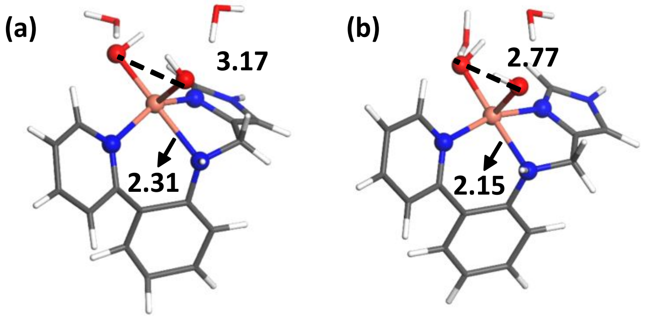

Figure S15 (a)The abstracted geometry of Cu(II)–$(OH)_2$ with two water molecules from CP2K after AIMD simulations and, (b) The geometry after reoptimization using a DFT method in Gaussian 16.

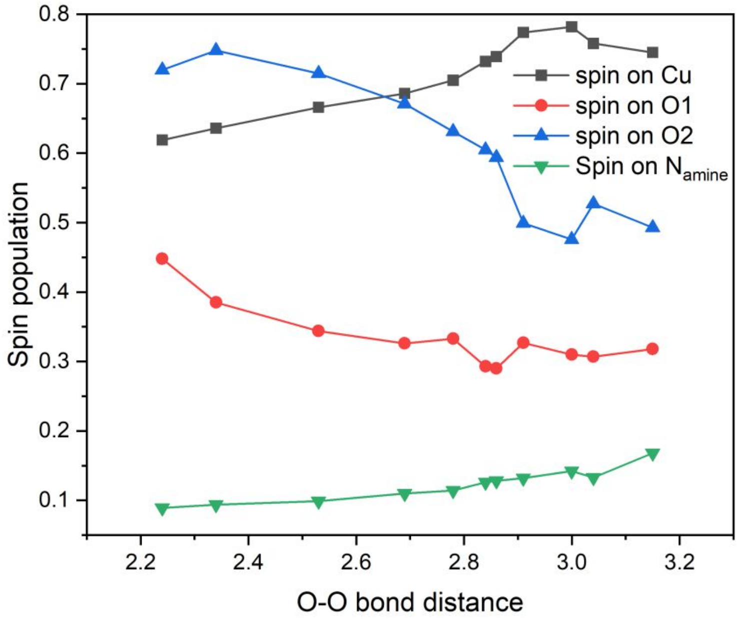

Figure S16 Changes in the spin populations on Cu, N_{amine}, and two O atoms from hydroxo groups with the increase in the O–O bond distance.

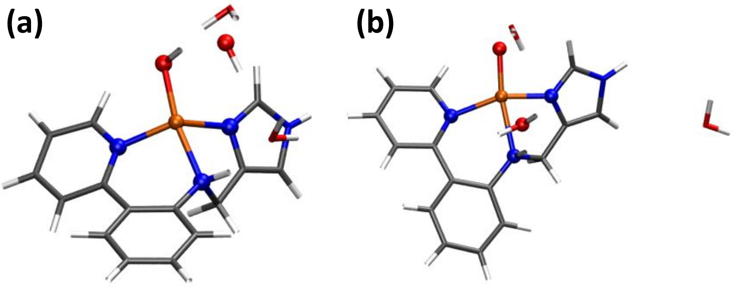

Figure S17 (a) Initial geometry of **T–12** optimized in Gaussian 16; (b) The abstracted geometry from CP2K after the AIMD simulations

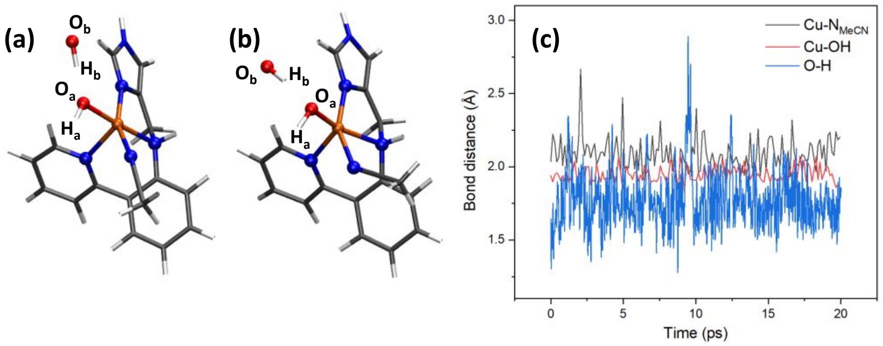

Figure S18 (a) The optimized $\mathbf{T_{MeCN}}$**–1** intermediate in Gaussian 16; (b) The abstracted geometry from CP2K after the AIMD simulations; (c) The evolution of the Cu–N_{py}, Cu–N_{IM}, Cu–N_{amine}, Cu–N_{MeCN}, Cu–OH, and Cu–O_aH_b bond distances in $\mathbf{T_{MeCN}}$**–1** intermediate with two water molecules along the NVT trajectory.

Table S5 Comparison of selected bond distances in the optimized $\mathbf{T_{MeCN}}$**–1** intermediate and the geometry obtained by AIMD, including the mean, minimum, and maximum values along the NVT trajectory.

	PBE0-D3/SVP, implicit MeCN solvent	PBE-D3/DZVP, explicit MeCN solvent		
		MEAN	MIN	MAX
Cu–N_{Py}	2.22	2.18	1.86	2.56
Cu–N_{IM}	2.02	2.05	1.84	2.36
Cu–N_{amine}	2.12	2.12	1.90	2.37
Cu–N_{MeCN}	2.06	2.09	1.84	2.73
Cu–OH	1.90	1.94	1.79	2.13
Cu–O_aH_b	1.51	1.77	1.28	2.89

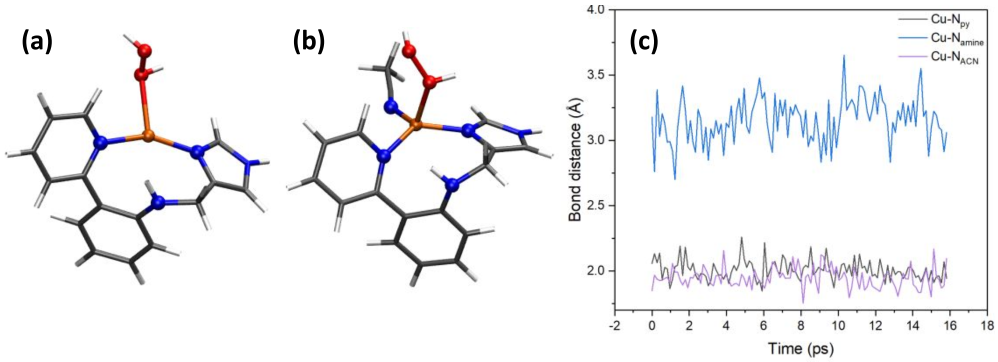

Figure S19 (a) The optimized **CSS-2** intermediate in Gaussian 16; (b) The abstracted geometry from CP2K after the AIMD simulations; (c) The evolution of the Cu–N_{py}, Cu–N_{IM}, Cu–N_{amine}, Cu–N_{MeCN}, and Cu–O_2H_2 bond distances in **CSS_{MeCN}–2** intermediate with two water molecules along the NVT trajectory.

Table S6 Comparison of selected bond distances in the optimized **CSS_{MeCN}–2** intermediate and the geometry obtained by AIMD, including the mean, minimum, and maximum values along the NVT trajectory.

	PBE0-D3/SVP, implicit MeCN solvent	PBE-D3/DZVP, explicit MeCN solvent		
		MEAN	MIN	MAX
Cu–N_{Py}	1.97	2.01	1.83	2.31
Cu–N_{IM}	1.94	2.01	1.83	2.26
Cu–N_{amine}	2.80	3.15	2.59	3.66
Cu–N_{MeCN}	5.40	1.96	1.75	2.37
Cu–O_2H_2	2.36	2.53	1.97	3.73

Table S7 The <S^2> values of intermediates and transition states shown in the energy profiles.

Intermediate	**OSS-TS1**	**OSS-1**	**T-1**	**T-2**	**T-TS1**
<S**2>	0.28	0.97	2.01	2.01	2.01
Intermediate	**T-3**	**T-4**	**T-TS2**	**T-5**	**T-6**
<S**2>	2.01	2.02	2.02	2.03	2.03
Intermediate	**T-7**	**T-TS3**	**T-8**	**T-10**	**OSS-TS2**
<S**2>	2.03	2.03	2.03	2.01	0.35
Intermediate	**T-11**	**T-TS5**	**T-12**	**OSS_{MeCN}-TS1**	**T_{MeCN}-1**
<S**2>	2.02	2.02	2.02	0.32	2.01
Intermediate	**T_{MeCN}-2**	**T_{MeCN}-TS1**	**T_{MeCN}-3**	**OSS_{MeCN}-TS1’**	**T_{MeCN}-1’**
<S**2>	2.01	2.01	2.01	0.37	2.01
Intermediate	**T_{MeCN}-2’**	**T_{MeCN}-TS1’**	**T_{MeCN}-3’**	**T_{MeCN}-4’**	
<S**2>	2.01	2.01	2.01	2.01	

From the above table, it can be observed that the <S^2> values of the triplet states are very close to 2.0, indicating low spin contamination. In contrast, the spin contamination in the open-shell singlets is high, particularly for the intermediates, as the <S^2> values approach 1.0. Notably, the transition states in the open-shell singlet state exhibit relatively low spin contamination, with <S^2> values ranging from 0.28 to 0.37. This suggests minimal contamination from triplet states, which is not enough to drive the <S^2> value closer to 1.0.
